# MDSINE: Microbial Dynamical Systems INference Engine for microbiome time-series analyses

**DOI:** 10.1186/s13059-016-0980-6

**Published:** 2016-06-03

**Authors:** Vanni Bucci, Belinda Tzen, Ning Li, Matt Simmons, Takeshi Tanoue, Elijah Bogart, Luxue Deng, Vladimir Yeliseyev, Mary L. Delaney, Qing Liu, Bernat Olle, Richard R. Stein, Kenya Honda, Lynn Bry, Georg K. Gerber

**Affiliations:** Department of Biology, Program in Biotechnology and Biomedical Engineering, University of Massachusetts Dartmouth, 285 Old Westport Road, N. Dartmouth, MA 02747 USA; Massachusetts Host-Microbiome Center, Department of Pathology, Brigham and Women’s Hospital, Harvard Medical School, 221 Longwood Ave, Boston, MA 02115 USA; RIKEN Center for Integrative Medical Sciences (IMS), Yokohama, Kanagawa 230-0045 Japan; Vedanta Biosciences, 501 Boylston Street, Suite 6102, Boston, MA 02116 USA; Department of Biostatistics and Computational Biology, Dana-Farber Cancer Institute, 450 Brookline Avenue, Boston, MA 02215 USA

## Abstract

Predicting dynamics of host-microbial ecosystems is crucial for the rational design of bacteriotherapies. We present MDSINE, a suite of algorithms for inferring dynamical systems models from microbiome time-series data and predicting temporal behaviors. Using simulated data, we demonstrate that MDSINE significantly outperforms the existing inference method. We then show MDSINE’s utility on two new gnotobiotic mice datasets, investigating infection with *Clostridium difficile* and an immune-modulatory probiotic. Using these datasets, we demonstrate new capabilities, including accurate forecasting of microbial dynamics, prediction of stable sub-communities that inhibit pathogen growth, and identification of bacteria most crucial to community integrity in response to perturbations.

**Electronic supplementary material:**

The online version of this article (doi:10.1186/s13059-016-0980-6) contains supplementary material, which is available to authorized users.

## Background

Increasing evidence indicates that the microbiota is critical to normal host physiology and a driver of human disease when it is disrupted to cause dysbiosis. The microbiota is inherently dynamic [1, 2], starting with successive colonization of the infant and demonstrating day-to-day variability in the healthy adult due to environmental and other factors. These dynamics are driven by networks of multispecies interactions that involve competition for limited nutrients and attachment sites [3], direct killing via bacteriocins [4], growth support through secretion of extracellular enzymes or metabolites that other species can cross-feed on [5], and a variety of other mechanisms.

Therapeutic manipulation of the microbiota is currently an area of intense investigation as a possible treatment for infectious [6], autoimmune, and other diseases. However, these efforts are hampered by limited methodologies for predicting dynamic behaviors of the microbiota when subjected to perturbations, including dietary changes, infections, and antibiotics. These perturbations can lead to dramatic shifts in microbial composition and even community collapse, which cannot be predicted without advances on existing computational analysis tools. Standard computational analysis methods for analyzing microbial community structures do not explicitly account for time-varying behavior, typically using correlational techniques [7] to find undirected connections among bacteria, which cannot be used to predict dynamic behaviors of the ecosystem.

Dynamical systems models provide an alternative and powerful framework for analyzing microbiome time-series data [8–10], with significant advantages over correlational techniques [7], including the capability to forecast future system behaviors, to characterize formal properties such as stability, and to infer directed and causal relationships [1, 2, 11]. With standard numerical integration techniques [12], however, these models are computationally intractable on large datasets. We previously presented an algorithm [8] that does not require extensive or explicit numerical integration for inferring parameters of a dynamical systems model, the generalized Lotka-Volterra (gLV) differential equations, and demonstrated for the first time the capability to accurately forecast gut microbiota dynamics in animal and human studies [8, 10]. The gLV model was subsequently successfully applied by other groups to analyzing microbiome time-series data [9, 13]. Despite these prior innovations, existing algorithms suffer from major shortcomings, including their inability to estimate confidence in predictions and their lack of models of the statistical properties of high-throughput microbiome sequencing data [14]; moreover, complete software packages have not been made available to the community.

**Fig. 1:**
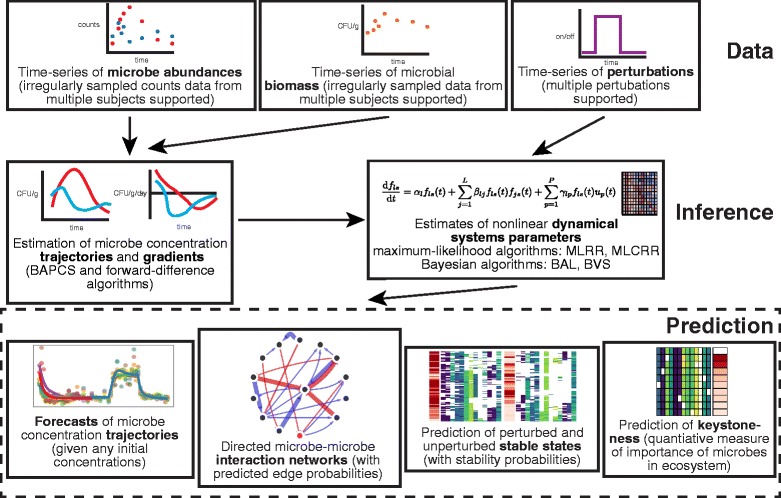
Schematic of the MDSINE software, which provides a comprehensive toolbox for dynamical systems analyses of microbiota time-series data. MDSINE implements a new algorithm, Bayesian Adaptive Penalized Counts Splines (*BAPCS*), for estimating microbial growth concentrations (trajectories) and their changes over time (gradients) from sequencing data; optionally, gradients can instead be estimated using our previously described first-order difference method. The software implements three new algorithms for dynamical systems inference: maximum likelihood constrained ridge regression (*MLCRR*), Bayesian adaptive lasso (*BAL*), and Bayesian variable selection (*BVS*). Our previously published method [8], the maximum likelihood unconstrained ridge regression algorithm (*MLRR*), is also implemented in MDSINE for comparison

Here we present the Microbial Dynamical Systems INference Engine (MDSINE), an open source software package that performs all analysis steps from reading data files through to the generation of figures (Fig. 1). MDSINE significantly extends our prior work by implementing: (a) a new technique for accurate estimation of microbial growth trajectories and gradients, specifically tailored for microbiome sequencing data that may be noisy and irregularly/sparsely sampled in time; (b) Bayesian [15] methods for estimation of confidence in parameters, including connectivity in microbial interaction networks; and (c) biologically realistic constraints on model parameters. The software package provides users with several alternative inference algorithms and analysis options, enabling both exploratory and focused analyses. In the remainder of this article, we describe the MDSINE algorithms, demonstrate the performance of MDSINE on data simulated to mimic key properties of real microbiome studies, and finally apply our method to analysis of two new in vivo experimental datasets, illustrating the utility of our method for predicting the dynamics of infection with an enteric pathogen and investigating the stability of a probiotic cocktail.

## Results

### Overview of algorithms implemented in the software

The MDSINE software (Fig. 1) provides a comprehensive toolbox for dynamical systems analyses of microbiota time-series data. MDSINE requires two inputs: (1) data measuring abundances of microbes over time in the ecosystems of interest, typically consisting of counts (e.g., high-throughput 16S rRNA sequencing data) that may be irregularly sampled temporally; and (2) data measuring overall microbial biomass over time in the ecosystem (e.g., universal 16S rRNA quantitative PCR (qPCR) data). The user may also optionally specify the temporal profiles of one or more experimental perturbations, such as a dietary change or antibiotic treatment. Note that data may include measurements for multiple subjects and subjects do not need to be sampled at the same time points. The same microbial taxa need not be present in all subjects, although a sufficient number of data points measuring dynamics of each taxon must be provided for accurate inference (see “Discussion” section).

MDSINE estimates microbial growth concentrations (trajectories) and their changes over time from the input data. These estimates are then used to infer the parameters of an extended gLV nonlinear ordinary differential equations model (see “Methods”). Inferred parameters are then used to make predictions, including forecasts of microbial growth trajectories given starting microbe concentrations specified by the user, directed microbe–microbe interaction and perturbation effect networks, formal stability analyses providing predictions of which combinations of microbes will stably coexist and at what concentrations, and keystoneness analyses providing a quantitative measure of the marginal importance of each species in the ecosystem.

In order to evaluate the performance of several alternative parameter inference approaches, we implemented four different algorithms in MDSINE (Additional file 1: Table S1). Two of the algorithms estimate model parameters using maximum likelihood-based techniques. The first of these algorithms, maximum likelihood ridge regression (MLRR) was previously presented by us [8]; it is the only scalable and documented published method for microbial dynamical systems inference but was yet to be included in a publicly available software package. The second of the maximum likelihood-based algorithms, maximum likelihood constrained ridge regression (MLCRR), is a novel extension of the MLRR method that constrains bacterial growth rates and self-inhibition terms in the model to biologically realistic settings and uses an efficient method for inference based on quadratic programming (see “Methods”).

We developed novel Bayesian inference algorithms that provide additional functionality that the maximum likelihood-based techniques do not, namely: (1) estimates of error in inferences of dynamical systems parameters; and (2) statistical modeling of high-throughput sequencing count-based data over time. The first feature, as we demonstrate below, is particularly important for interpreting the relevance of microbial interactions and predictions of dynamics inferred from real microbiome time-series datasets. The second feature builds on substantial work by others demonstrating that greater accuracy can be achieved in analyses of high-throughput sequencing data by directly modeling count data [14, 16]. Our approach, Bayesian Adaptive Penalized Counts Splines (BAPCS), uses a method similar to that employed by DESeq2 [14] but extended for time-series data and allowing for irregular temporal sampling. The BAPCS algorithm is used to denoise the input microbiome time-series data, estimating the underlying growth trajectories and their gradients. These estimates then serve as input into two alternative Bayesian dynamical systems inference algorithms. The first of the two Bayesian inference algorithms, Bayesian Adaptive Lasso (BAL), is a regularization-based approach conceptually similar to the maximum likelihood ridge regression-based algorithms (MLRR and MLCRR) described above, but can: (a) potentially discriminate true interactions from noise better because it provides more shrinkage for coefficients that are close to zero [17]; (b) is more flexible, as it allows for different degrees of regularization on each interaction coefficient [9]; and (c) is fully Bayesian, providing estimates of variability for all parameters in the model. The second of the two Bayesian inference algorithms, Bayesian Variable Selection (BVS), uses an alternative approach to the regularization-based methods (MLRR, MLCRR, and BAL) by directly modeling the presence/absence of microbe–microbe interactions or microbe–perturbation effects. We were interested in evaluating a variable selection approach against the regularization-based methods, as variable selection techniques have been shown to reduce bias in model estimates in many settings [18]. Moreover, our BVS algorithm allows for direct inference of the underlying qualitative network of bacterial interactions that is of biological interest. See “Methods” and Additional file 2 for a complete description of the Bayesian algorithms.

### Validation on simulated ground-truth data

Ground-truth information is necessary for fully benchmarking the inferential and predictive capabilities of an algorithm. However, the availability of such data is currently too limited for microbial dynamical systems. Thus, we simulated data from an in silico microbial dynamical systems model that captures key features of real microbiome experiments to benchmark and validate MDSINE (see “Methods” for complete details). Briefly, we mimicked key features of real microbiome time-series data including noisy count-based measurements with different sequencing depths and assuming compositionality of data [19, 20], variability among subjects, limited temporal sampling resolutions, and a pathogen being introduced into a pre-existing microbiota. Repeated random sampling was used to generate different initial conditions and to add noise to the simulated measurements. The simulated data were then used to evaluate several metrics of algorithm performance, including: (a) accuracy of growth rates and interaction coefficients estimates; (b) accuracy of inference of presence/absence of microbial interactions (network structure); and (c) accuracy of prediction of microbial concentrations over time given initial conditions not previously seen by the algorithm.

**Fig. 2:**
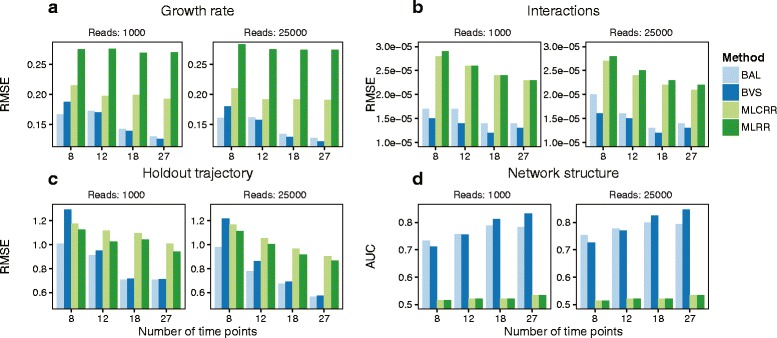
New inference algorithms in MDSINE outperform our previously published method on simulated data. Data were simulated to capture key features of real microbiome surveys, including noise and compositionality. Simulations assumed an underlying dynamical systems model with ten species observed over 30 days and an invading species at day 10. The number of time points sampled was varied between 8 and 27 to mimic common experimental designs and sequencing depths of 1000 or 25,000 reads were evaluated. Performance of the four MDSINE inference algorithms, maximum likelihood ridge regression (MLRR), maximum likelihood constrained ridge regression (MLCRR), Bayesian adaptive lasso (BAL), and Bayesian variable selection (BVS), were compared. Algorithm performance was assessed using four different metrics: root mean square error (RMSE) for microbial growth rates (**a**); RMSE for microbial interaction parameters (**b**); RMSE for prediction of microbe trajectories on held-out subjects given only initial microbe concentrations for the held-out subject (**c**); and area under the receiver operator curve (AUC ROC) for the underlying microbial interaction network (**d**). Lower RMSE values indicate superior performance, whereas higher AUC ROC values indicate superior performance

Overall, the three new MDSINE algorithms (MLCRR, BAL, and BVS) outperformed our previous method (MLCRR) on all metrics (Fig. 2). The two Bayesian algorithms (BAL and BVS) showed the greatest robustness to lower sequencing depths and lower resolutions of temporal sampling and demonstrated particularly strong performance on inferring microbial interaction parameters and the underlying network. In contrast, both MLRR and MLCRR as well as standard Spearman correlation analyses (area under the curve (AUC) of 0.53 in the best case) had very poor performance on the underlying network inference task for all simulation regimes (Fig. 2d).

Performance on the microbial concentration prediction task was most affected by sequencing depth and temporal sampling resolution for all the MDSINE algorithms, whereas estimation of growth rates and interaction coefficients were less affected. To investigate the effects of read depth further, we performed simulations over a range of read depths from 200 sequences per sample (very suboptimal) to 25,000 sequences per sample. These simulations (Additional file 3: Figure S1) showed that performance on all metrics, and in particular the microbial concentration prediction metric, did improve with greater read depths for all MDSINE algorithms but essentially saturated at 5000 sequences per sample. These results suggest that the MDSINE inference procedures are robust to compositionality constraints in data and, moreover, maintain robustness even at low sequencing depths. This robustness may in part be due to MDSINE’s capability to leverage continuity over time to compensate for noise in data.

We also investigated whether connectivity (in-degree) or growth rates of the simulated taxa affected the microbial concentration prediction task. No clear relationship was evident in the case of in-degree. Positive correlations between growth rates of taxa and the ability to forecast their concentrations were seen for all the MDSINE algorithms, suggesting that the algorithms may predict trajectories of faster-growing bacteria with more accuracy in some ecosystems. However, these correlations did not reach statistical significance (Spearman correlation, *p* values for BAL, BVS, MLRR, and MLCRR of 0.30, 0.35, 0.33, and 0.30, respectively).

### Validation against new experimental datasets

We next validated the performance of MDSINE on two new experimental datasets with several necessary or desirable properties for algorithm evaluation. First, frequent temporal sampling (26–56 time points) was performed to support accurate inference of dynamics [2]. Second, microbial ecosystems were perturbed rather than just observed over time. Third, concentrations of bacteria were measured rather than just relative abundances; these measurements are particularly important for studying infections and other perturbations that may alter the bacterial biomass [1, 2]. Finally, although not essential for application of our method, we used gnotobiotic experimental systems to ensure that strains could be tracked unequivocally in vivo over time.

### *Clostridium difficile* infection experiments

Our first experimental dataset evaluated the dynamics of infection with *Clostridium difficile* [10] (Additional file 4: Figure S2), a pathogen that causes significant morbidity and mortality in humans. We employed a gnotobiotic mouse model in which animals were pre-colonized with human commensal bacterial type strains, termed the GnotoComplex microflora, chosen to capture the phylogenetic diversity and key physiologic capabilities of a native gut microflora, including metabolism of complex polysaccharides and bile acid transformations (see Additional file 1: Table S2 and “Methods” for details). After allowing 28 days for the commensal microbiota to establish, we infected mice with *C. difficile* spores and monitored the animals for an additional 28 days. Mice had diarrhea and showed signs of moderate distress within 3 days of *C. difficile* infection but all exhibited full clinical recovery within 10 days. Throughout the 56-day experiment, 26 fecal samples per mouse were collected and interrogated via high-throughput 16S rRNA sequencing to determine abundances of species and 16S rRNA qPCR using universal primers to estimate the total bacterial biomass present.

The temporal sampling scheme for this experiment was designed based on our prior experience with kinetics of enteric infections in mouse models [21] and response of the microbiota to perturbations [22, 23]. Our analyses of these prior studies using our MC-TIMME algorithm indicated that OTUs that change in abundance after a perturbation will generally first show detectable changes within a window of 1–3 days and reach steady-state within 2 weeks. Thus, our general approach to designing time-series studies of the microbiota, subject to economic and logistic constraints that prevent sampling every day over the entire time course, is to sample at least daily for 4 days post-perturbation, then reduce sampling to every 2 days up to 2 weeks post-perturbation, and then reduce sampling to every 3–4 days thereafter until the next perturbation (if any).

We assessed predictive performance of MDSINE on the *C. difficile* infection data using a hold-one-subject-out cross-validation procedure. In this procedure, MDSINE was run on all data from all but one of the mice (the held-out subject) and model parameters were inferred. Using the inferred model parameters and the measured concentrations of the microbiota at an initial time point for the held-out mouse, the trajectory of concentrations of *C. difficile* for that mouse was then forecast for all the remaining time points using numerical integration. This procedure was repeated for each mouse in turn and predictive performance was evaluated as the root mean square error (RMSE) between the predicted trajectory and the actual data (see “Methods” for details). The new algorithms in MDSINE outperformed our previous method on this task, with the Bayesian algorithms showing the strongest performance (Additional file 1: Table S3) with RMSEs of 0.56 and 0.66 colony forming units (CFUs)/g stool. These error rates in predictions, corresponding to about a half log in CFU/g, are close to variability in measurements we have observed with traditional culture-based techniques and due to differences in sample handling [21] and thus represent strong predictive performance in terms of realistic biological systems.

**Fig. 3:**
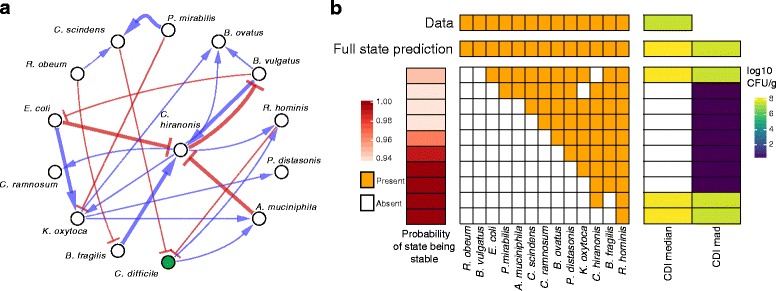
Application of MDSINE to an experimental dataset evaluating the dynamics of *Clostridium difficile* infection in gnotobiotic mice. Germ-free mice were pre-colonized with the GnotoComplex microflora, a mixture of human commensal bacterial type strains chosen to capture the phylogenetic diversity and key physiologic capabilities of a native gut microflora. After the commensal microbiota were allowed to establish for 28 days, mice were infected with *C. difficile* spores and monitored for an additional 28 days. Throughout the experiment, 26 fecal samples per mouse were collected and interrogated via high-throughput 16S rRNA sequencing to determine abundances of species and 16S rRNA qPCR using universal primers to estimate the total bacterial biomass present. **a** Predicted directed microbe–microbe interaction network. *Edge thickness* denotes the magnitude of the evidence favoring presence of the interaction (Bayes factors [35]); only edges with strong evidence (Bayes factor ≥10) are displayed. **b** Predicted stable combinations of strains for each possible size of sub-community that optimally inhibit *C. difficile* colonization. Each *row* depicts the sub-community (combination of commensal strains) of a given size that is predicted to stably colonize the gut in the absence of the pathogen and is predicted to maximally inhibit *C. difficile* infection at the experimental end point (28 days). *CDI median* predicted median concentration of *C. difficile* at 28 days, *CDI mad* predicted absolute deviation of the median of the *C. difficile* concentration at 28 days

We next used MDSINE to infer the underlying qualitative network of microbe–microbe interactions from the complete dataset (Fig. 3a). The resulting network strongly predicts the presence of 23 interactions among species (Bayes factor ≥10), including inhibition of *C. difficile* by *Clostridium scindens* and *Roseburia hominis*. A *C. scindens* inhibitory effect against *C. difficile* mediated by the alteration of host bile acid composition has previously been demonstrated in conventional mice [10], providing confirmatory evidence that MDSINE can detect causal interactions from longitudinal microbiota data.

In order to predict which bacterial compositions optimally confer resistance against *C. difficile* infection, we used MDSINE to predict the stability and *C. difficile* inhibitory capacity of all subsets of the commensal strains (Fig. 3b). We began by calculating which of the 2^13^ − 1 possible bacterial combinations were predicted to have biologically meaningful steady-state concentrations (non-negative values). Steady states with a probability of stability >90 % were further analyzed using numerical integration starting with the predicted concentrations and adding a simulated *C. difficile* challenge of 10^5^ CFU/g, corresponding to a high infectious dose (see “Methods” for details). Combinations of bacteria displayed in Fig. 3b represent those (for all strains in the defined microbiota down to one strain) that led to the minimal median predicted concentrations of *C. difficile* at 28 days, the duration of our actual experiment. Intriguingly, MDSINE predicted that the smallest stable sub-community capable of excluding *C. difficile* with probability >90 % requires just three organisms (*R. hominis*, *B. fragilis*, and *C. hiranonis*). Interestingly, although MDSINE predicts similar colonization resistance for larger communities (all of them including the stable three-species sub-community), the stability probability decreases somewhat for the larger communities.

It is of interest to understand whether bacterial biomass data are necessary for inference of bacterial interaction networks, or at least if less frequent biomass measurements would suffice, given that biomass has not been routinely measured in many microbiome studies. To test the necessity of biomass data for the *C. difficile* experiments, we replaced biomass measurements with the average value across all mice and all time points, hence providing no useful information on biomass to MDSINE. This “no biomass” inference resulted in an AUC value of 0.76 when compared with the “reference” network inferred using all biomass data. The “no biomass network” had no incoming edges for *C. difficile* with strong evidence (Bayes factors ≥10) but edges for *C. scindens* and *R. hominis* interactions still had borderline Bayes factors of 7.7 and 3.0, respectively. We additionally tested whether our BAPCS algorithm could be used to improve network inference, by interpolating bacterial biomass values, in the scenario in which bacterial biomass was sampled less frequently than bacterial relative abundances. For this test, we hid either six or ten biomass measurements, with time points chosen using the scheme discussed above that prioritizes sampling around perturbations. When six biomass measurements were hidden, the inferred bacterial interaction network was almost identical to the “reference” network (AUC = 0.93), whereas when ten biomass measurements were hidden, performance degraded to the level of the “no biomass” network (AUC = 0.74). These results suggest that many edges of the bacterial interaction network can still be accurately inferred in the absence of biomass data but that biomass data do provide important additional information. Further, our method can be used to interpolate biomass measurements to some extent, allowing for less frequent biomass sampling, but performance dropped off dramatically beyond about 25 % missing measurements.

#### Probiotic cocktail colonization and stability experiments

Our second experimental dataset evaluated the dynamics of colonization of gnotobiotic mice with a probiotic bacterial cocktail and subsequent effects of a dietary perturbation (Additional file 5: Figure S3). We recently described a set of Clostridia strains (VE-202) that are potent inducers of regulatory T cells (Tregs) and suppressors of inflammation [24] and are now being investigated for treatment of inflammatory bowl diseases. To characterize stability and robustness properties of the VE-202 cocktail, we colonized germ-free mice with 13 members of the cocktail and alternated mice between a standard high-fiber diet and a low-fiber dietary perturbation. These dietary shifts were enforced for two reasons. First, accurate model inference requires a sufficient number of non-equilibrium observations, which we expected to achieve with a short dietary perturbation per our previous work demonstrating broad responses to dietary shifts in the microbiota with taxon-dependent kinetics [22]. Second, we were interested in whether abundances of the probiotic cocktail organisms would be appreciably shifted with a change to a low-fiber diet, which more closely mimics aspects of a human diet.

Over the nine-week experiment, 56 fecal samples per mouse were collected (daily sampling with some exceptions for logistical reasons). Bacterial concentrations were measured using strain-specific qPCR primers to estimate concentrations of the microbes (see “Methods”; Additional file 1: Table S4). Note that for these experiments total bacterial biomass was not measured independently, unlike for the *C. difficile* infection experiments that combined taxa relative abundance data from 16S rRNA amplicon sequencing with total bacterial biomass estimated using 16S rRNA universal primers.

**Fig. 4:**
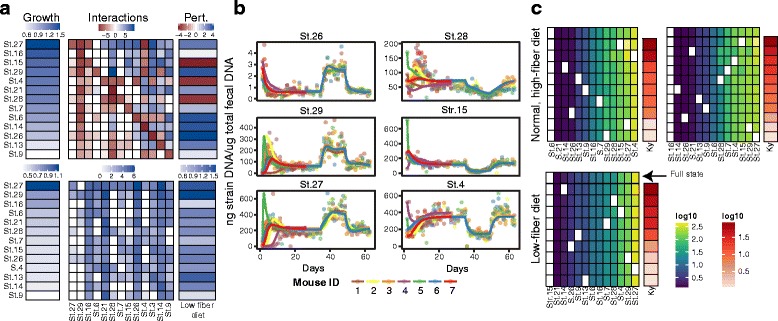
Application of MDSINE to an experimental dataset evaluating stability of a probiotic cocktail in gnotobiotic mice. Germ-free mice were inoculated with 13 Clostridia strains from the VE-202 cocktail, a mixture of bacteria previously shown to be Treg inducers [24]. Over the 9-week experiment, mice were alternated between a standard high-fiber diet and a low-fiber dietary perturbation. We collected 56 fecal samples per mouse and these were interrogated using strain-specific qPCR primers to estimate strain concentrations. **a** Example of inferred growth and interaction parameters and their variability. The *top grid* displays mean parameter estimates and the *bottom grid* displays standard deviations of parameter estimates. Strains are ordered by their mean estimated growth rates on the standard diet. *Pert.* perturbation effect, *St.* strain. **b** Example forecasts of microbial concentration trajectories. Forecasts were obtained using a hold-one-subject-out procedure. Briefly, MDSINE was run on all data from all but one of the mice (the held-out subject) and model parameters were inferred. Using the inferred model parameters (including for the perturbation) and the measured concentrations of the microbiota at an initial time point for the held-out mouse, the trajectories of the microbiota for the held-out mouse were then forecast for all the remaining time points; the procedure was repeated for each mouse. *Solid lines* denote predicted trajectories and *circles* denote data. **c** Keystoneness analyses for high-fiber (*top*) and low-fiber (*bottom*) diets. *Rows* represent all possible stable states in which each strain has been removed in turn and the others are present (if that configuration is stable). The *grid* displays predicted steady-state concentrations of strains (log_10_ ng strain DNA/μg total fecal DNA), with *white entries* indicating absent strains. *Ky* keystoneness, a measure assessing the marginal predicted quantitative effect of removing each strain from the full community, with larger values indicating greater effects on the overall ecosystem

We again evaluated the ability of MDSINE to predict microbial growth concentrations using a hold-one-subject-out cross-validation procedure. In this procedure, MDSINE was run on all data from all but one of the mice (the held-out subject) and model parameters were inferred. Using the inferred model parameters (including for the applied dietary perturbation) and the measured concentrations of the microbiota at an initial time point for the held-out mouse, the trajectories of concentrations of the microbiota for that mouse were then forecast for all the remaining time points using numerical integration. Figure 4a, b and Additional file 6: Figure S4 display examples of inferred parameter distributions and predicted trajectories. Interestingly, for this dataset we found no significant differences between the new MDSINE algorithms and our prior algorithm with respect to prediction error (Table S3).

We used MDSINE to predict the contribution of each member of our bacterial probiotic cocktail to the maintenance of stability and community structure in the presence and absence of the dietary perturbation. Intriguingly, we found that while the low-fiber diet clearly alters the concentrations of many strains in the cocktail, this perturbation does not significantly change the overall stable biodiversity profile of the cocktail (Additional file 7: Figure S5). To quantify the importance of each strain in maintaining stable community structure, we applied a new measure that we term keystoneness (Fig. 4c). This measure assesses the marginal predicted quantitative effect of removing each strain from a bacterial community (see “Methods” for details). On the high-fiber diet, strain 15 (closest known taxon, *Clostridium asparagiforme*) exhibited the largest keystoneness value (Fig. 4c, top). Interestingly, MDSINE predicts that, for the low-fiber diet, strain 15 cannot be present if the community is to remain stable and strain 14 (closest known taxon, *Ruminococcus* sp. ID8) attains the highest keystoneness value (Fig. 4c, bottom). We note that strains 14 and 15 do not have significantly greater connectivity in the inferred qualitative network structure (Additional file 8: Figure S6), suggesting that the central roles of these species would not be uncovered using standard qualitative measures for finding keystone species [13].

## Discussion

Studies of temporal dynamics of the microbiota coupled to experimental interventions are essential to move beyond descriptive associations to causal mechanisms and, ultimately, to rationally design approaches to manipulate the microbiota to achieve precise and durable therapeutic benefits. However, analysis of data from such studies has been hampered by a lack of appropriate computational tools. In this work, building on our previous research, we present a set of new algorithms implemented in an open-source, actively maintained software package that allows users to infer dynamical systems models from high-throughput microbiome data and generate precise quantitative predictions about the temporal dynamics and stability of the microbial ecosystems.

To showcase the potential of our method for generating quantitative predictions and testable hypotheses in the context of enteric infections and probiotic cocktail design, we applied our framework to two new datasets generated in our labs. For the first dataset, we conducted massive simulations to determine microbiota combinations predicted to maximize inhibition of the pathogen, *C. difficile*. MDSINE predicted that a community as small as three species, *R. hominis*, *B. fragilis*, and *C. hiranonis*, has the capacity to provide colonization resistance against *C. difficile.* This result highlights the importance of considering quantitative and indirect effects in microbial interaction networks [25] as only *R. hominis* is predicted to directly inhibit *C. difficile* whereas the other species in the sub-community are predicted to indirectly support growth of *R. hominis* (Fig. 3a). Interestingly, MDSINE predicts that larger communities have somewhat lower stability probabilities than the three-species community, suggesting that larger communities may have greater numbers of destabilizing interactions in the network. Overall, these results, which suggest new strategies for rational design of bacteriotherapies [6] for *C. difficile* infection, demonstrate the unique probabilistic and quantitative predictive capabilities of the MDSINE framework.

For our second experimental dataset, we evaluated the stability to dietary changes of a probiotic commensal bacterial cocktail under investigation for treatment of inflammatory bowel disease (IBD). Diet is an acknowledged but poorly defined modulator in IBD, with dietary factors directly interacting with both epithelial and immune cells and indirectly affecting immune homeostasis by modulating the intestinal microbiota [26]. Moreover, dietary treatments for IBD are often recommended by clinicians or self-initiated by patients [27]. For these reasons, any probiotic cocktail for IBD treatment must be capable of withstanding changing dietary regimes. Application of MDSINE to data from our gnotobiotic mouse model measuring changes in concentrations of strains in a probiotic cocktail subjected to dietary shifts intriguingly suggests different ecological roles of the strains in maintaining community structure depending on the dietary context. These results highlight the complexity of probiotic design and showcase the unique capabilities of MDSINE for this application to exploit dynamical systems methods to tackle a per se combinational design problem to efficiently and rationally choose among probiotic compositions.

On both simulated and real experimental data, our new algorithms generally outperformed our previous method. Our new algorithms all constrain model parameters to biologically realistic values (non-negative growth rates and negative self-regulation effects), which accounts for some of the improvements in performance, particularly between the MLCR and MLCRR algorithms that differ only in this aspect. The algorithms also handled compositionality of data well, even with quite low read depths, which is likely due in part to their ability to exploit time-series information fully to estimate microbial populations (e.g., temporally adjacent samples can compensate for error), in contrast to standard static analysis methods that effectively treat each sample separately.

The Bayesian algorithms generally exhibited superior performance, likely due to a combination of their directly modeling noisy count data and their using inference methods that help to reduce bias in parameter estimates. Additionally, we found that the Bayesian methods provided much more reliable estimates of variability in parameters, particularly for the task of inferring underlying bacterial interaction networks. In principle, parameter variability can be estimated within maximum likelihood-based frameworks (Additional file 2), but we found these estimates to be quite poor. Interestingly, on one task, prediction of held-out trajectories for our probiotic stability dataset, the Bayesian algorithms did not have significantly better performance than the other methods. This dataset had very dense (daily or every other day) temporal sampling and used qPCR measurements to quantify strain concentrations. As discussed, the Bayesian methods were specifically designed to handle irregularly and sparsely temporally sampled sequencing count data and so apparently lost some of their advantages when these features were not present. However, the capability of the Bayesian methods to estimate parameter variability for this dataset was still important, as demonstrated with the keystoneness/stability results that use this information to substantially limit the number of states considered.

MDSINE models concentrations of micro-organisms rather than relative abundances, which requires estimates of the total microbial biomass in the ecosystem under study. In our *C. difficile* infection or probiotic cocktail experiments, we estimated total bacterial biomass via qPCR either with 16S rRNA universal primers or with strain-specific primers. These methods are susceptible to primer bias and other PCR-related issues; alternative methods, such as flow cytometry [28], could overcome some of these difficulties. However, most existing microbiome studies do not include any measurement of total microbial biomass. Can MDSINE still be applied to these data? In some cases, total microbial biomass is unlikely to change appreciably over the course of a study, such as in adult populations undergoing mild dietary shifts. For these cases, MDSINE can be run on just relative abundance data (e.g., 16S rRNA amplicon sequencing data) with the assumption that biomass is constant. In other cases, such as studies of initial colonization of the gut, total microbial biomass is assuredly changing and the assumption of constant total microbial biomass would be erroneous. For other studies, such as antibiotic exposures, infections, or major dietary shifts, the effect on total microbial biomass is not clear a priori. For our *C. difficile* infection dataset, as described in the “Results” section, many bacterial interactions could still be inferred under the assumption of constant bacterial biomass, but some key interactions were lost or evidence of their existence was substantially weakened. For these reasons, we would advocate for measuring total bacterial biomass in future longitudinal microbiome studies, at least in pilot phases, to determine whether biomass changes significantly.

In terms of runtime, the algorithms in MDSINE can feasibly scale to ecosystems with considerably more taxa than analyzed in the present work because they approximate the dynamical systems inference problem with linear systems of equations. The challenge of scaling to more complex ecosystems, therefore, is not primarily computational but is limited by data: an ecosystem with *N* taxa has *N*^*2*^*–N* potential interactions, requiring *O(N*^*2*^*)* informative data points to reliably estimate parameters in the absence of regularization or sparsity assumptions. Regularization and sparsity techniques implemented in MDSINE reduce these data requirements, as demonstrated in our simulation studies showing negligible loss of inference accuracy on key metrics even with >50 % data reduction. However, it is important to understand that measurements must be obtained under perturbations of the ecosystem, as multiple measurements at the same steady-state value will not contribute multiple highly informative data points [2]. For these reasons, as a broad guideline and assuming reductions in data requirements from regularization or sparsity assumptions as described, we recommend studies include perturbations and collect a minimum of *N*^*2*^/2 total data points (e.g., for a study with five subjects and 13 OTUs to be modeled, at least 17 time points per subject). For users of MDSINE studying complex microbial ecosystems with larger numbers of OTUs, we suggest modeling a smaller number of key OTUs or higher taxonomic groups (e.g., 10–20); we and others have successfully demonstrated this approach using the gLV model in prior publications [8–10].

Our approach has several limitations that suggest areas for future work. First, the underlying gLV dynamical systems model for MDSINE captures only pairwise microbe–microbe interactions and the effects of defined perturbations with relatively simple kinetics. Despite these limitations, we and others have demonstrated the surprisingly good performance of this model for analyzing complex host–microbial ecosystems [8–10, 13]. Moreover, the inference framework for MDSINE is quite general and could in the future support richer models of dynamics that incorporate factors such as host immune responses, metabolites, environmental effects, and nonparametric models of dynamics. Second, our method relies on a series of approximations to the underlying dynamical systems model. Our simulation studies demonstrate that this approximation generally works well; however, techniques to further improve these approximations, such as the use of Gaussian Processes [29], are an interesting area for future work on MDSINE. Third, we demonstrated application of MDSINE to gnotobiotic animal datasets with microbiota of limited diversity. As discussed, these datasets provided important features that facilitated evaluation of our algorithms and interpretability of the results. However, the microbiota of humans and conventional animals are considerably more complex and present additional analytical challenges. Extensions to MDSINE to automatically reduce the complexity of these ecosystems—for instance, by grouping together OTUs with similar dynamics while still maintaining predictive accuracy—is an interesting direction for future research. Finally, our experimental datasets rely on fecal sampling, which may not accurately reflect microbial populations in the gut, in particular for species that predominantly reside in regions upstream of the colon [21]. Measurements of total bacterial biomass from fecal samples also may not be representative of biomass in the gut if the density of stool changes significantly throughout the experiment, such as with severe diarrhea. There is also increasing recognition of the importance of the spatial organization of the microbiota (e.g., [30]) that will not be reflected in fecal samples. Future studies that obtain longitudinal samples of tissues in different regions of the gut will be important to more fully model and understand these incredibly rich ecosystems.

## Conclusions

MDSINE provides a new toolbox that will have broad applicability to studies of microbial dynamics, enabling robust analyses based on quantitative predictions and formal dynamical systems theory as demonstrated on our simulated and new experimental validation datasets. Our open-source software package implements new methods that not only outperform previous approaches but also provides novel functionality, including capabilities to estimate confidence in model parameters and predicted dynamics. Application of MDSINE to two new gnotobiotic experimental datasets demonstrates the capability to generate predictive hypotheses that standard microbiome analysis methods cannot and, moreover, suggests new strategies for rational design of bacteriotherapies.

## Methods

### Software availability

The Microbial Dynamical Systems INference Engine (MDSINE) is available as an open-source package including MATLAB source code and standalone executables for Mac OS X, Linux and Windows. The software reads formatted input data (strain counts table, total bacterial biomass measurements, and relevant metadata; see the repository instructions and manual for further details—links provided in the “Availability of data and materials” section), then infers dynamical systems models. Further analyses using the inferred models are supported in MDSINE, including predictions of qualitative interaction networks, trajectories, stable states, and keystoneness. R-utility scripts are provided in the package for visualizations of these analyses.

### Mathematical model overview

We use the extended generalized Lotka-Volterra (gLV) equations as previously described [8] for the underlying model of microbial dynamics. For the gLV model for *L* OTUs measured in *S* subjects, the rate of change of the concentration of OTU *l* in subject *s* is expressed as:

$$ \frac{d{f}_{ls}}{dt}={\alpha}_l{f}_{ls}(t)+{\displaystyle \sum_{j=1}^L{\beta}_{lj}{f}_{ls}(t){f}_{js}(t)+{\displaystyle \sum_{p=1}^P{\gamma}_{lp}{f}_{ls}(t)}}{u}_p(t) $$

The *α* parameters represent unbounded growth rates, the *β* parameters represent pair-wise microbe-microbe interactions, and the *γ* parameters represent effects of *P* perturbations. The functions *u*_*p*_*(t)* are binary-valued, indicating if the given perturbation is present at time *t*. Unlike in our previous approach [8], which did not assume constraints on the parameters, here we assume positive growth rates and negative self-interaction rates, i.e., *α* > 0 and *β*_*ll*_ < 0. These constraints enforce the biologically realistic assumption of logistic growth in the absence of interactions, i.e., growth up to a finite carrying capacity of the ecological system.

We use a “gradient matching” approach to estimate the ODE parameters. The concept underlying this approach is that if estimates of the gradient and trajectory values are available, parameters can be estimated by solution of systems of equations rather than systems of differential equations. In the case of the gLV model, the “gradient matching” system of equations is linear, and can be written as:

$$ \frac{d{f}_{ls}}{dt}\approx {\widehat{f}}_{lst}^{\prime}\approx {\alpha}_l{\widehat{f}}_{lst}+{\displaystyle \sum_{j=1}^L{\beta}_{lj}{\widehat{f}}_{lst}{\widehat{f}}_{jst}+{\displaystyle \sum_{p=1}^P{\gamma}_{lp}{\widehat{f}}_{lst}}}{u}_p(t) $$

Here, ${\widehat{f}}_{lst}$ represents an estimate of the concentration of OTU *l* in subject *s* at time point *t* and ${\widehat{f}}_{lst}^{\prime }$ represents the corresponding gradient estimate. These estimates are derived from data as described in the sections below and detailed in Additional file 2. We assume we have measurements of counts *Y*_*lst*_ (i.e., obtained via 16S rRNA sequencing) for each OTU *l* in subject *s* at time point *t*, where there are *L* OTUs, *S* subjects and *N*_*s*_ time points per subject. We also assume we have measurements of total bacterial biomass *W*_*st*_ (e.g., obtained via qPCR) for each time point *t* in each subject *s*.

The reduction of the gLV differential equations to a linear system of equations via the “gradient matching” approach enables application of statistical models for linear regression. However, we are still faced with estimating *L*^*2*^ *+ L + LP* parameters, which will result in an under-determined system for typical datasets. We thus developed several algorithms that use regularization or variable selection techniques during the parameter inference process. These algorithms are described below and detailed in Additional file 2.

#### Maximum-likelihood constrained ridge regression (MLCRR) algorithm

The overall objective of the MLCRR algorithm is to infer point-estimates of growth, interaction, and perturbation effect parameters for the gLV model from high-throughput 16S rRNA gene sequencing data and measurements of microbial biomass. As described above, typical microbiome time-series datasets will not have enough observations relative to potential microbe–microbe interactions and perturbation effects, resulting in an under-determined system. We solve this problem by employing an *L*_*2*_-regularization approach (also known as Tikhonov regularization or ridge regression [31]) with cross-validation to set the regularization parameters. The MLCRR algorithm extends our prior maximum likelihood-based algorithm MLRR [8] to constrain growth rates to positive values and self-interaction parameters to negative values. We developed an efficient quadratic programming-based approach for inferring parameters in the constrained problem, which is detailed in Additional file 2.

#### Bayesian algorithms

The overall objective of the MDSINE Bayesian algorithms is to infer distributions of growth, interaction, and perturbation-effect parameters for the gLV model from high-throughput 16S rRNA gene sequencing data and measurements of microbial biomass. Inferred distributions of parameters are then used to compute summary measures, including median predicted trajectories for OTUs, as well as measures of variability and strength of model selection evidence. As with the MLCRR maximum likelihood-based method, we use a “gradient matching” approach to infer parameters for the gLV model.

For the MDSINE Bayesian algorithms, we use a two-step estimation procedure. First, we estimate trajectories and gradients of OTU concentrations directly from sequencing counts and biomass data using our Bayesian Adaptive Penalized Counts Splines (BAPCS) method described below. Then, we use the estimated trajectories and gradients to infer the gLV parameters using Bayesian linear modeling techniques, Bayesian Adaptive Lasso (BAL) or Bayesian Variable Selection (BVS), each described below. For both steps, we use custom Markov Chain Monte Carlo (MCMC) algorithms detailed in Additional file 2.

The Bayesian Adaptive Penalized Counts Splines (BAPCS) algorithm in MDSINE estimates OTU concentration trajectories and gradients from data consisting of time series of noisy counts. Our formulation of the problem explicitly models differing numbers of sequencing reads between samples, overdispersion of counts, and irregular temporal sampling. We assume that counts data follow the Negative Binomial Distribution (NBD), which we and others have previously employed for modeling microbial sequencing counts data [16, 23]. For modeling the NBD dispersion parameter, we employ a method similar to that used by DESeq2 [14] but extended for time-series data. We model the continuous-time trajectory function for the NBD mean for each OTU in each subject using cubic B-splines [32], which provide an efficient and flexible framework for modeling time-series data that we and others have demonstrated yield accurate predictions for large-scale longitudinal biological datasets [33]. A challenge with B-spline methods is specifying the position and number of basis functions (break points); these choices influence the amount of smoothing applied to the data. To overcome this challenge, we use a penalized spline framework [32] with an adaptive Bayesian regularization approach. Our approach places the break points uniformly at a high frequency (default of every 2 days) and then adaptively regularizes the spline coefficients in nearby temporal regions to encourage minimal changes in the trajectory over time unless larger deviations are warranted by the data. To achieve this, we use a hierarchical adaptive Bayesian lasso-style [17, 34] model. See Additional file 2 for a complete description of the BAPCS model and inference algorithm.

The Bayesian Adaptive Lasso (BAL) algorithm in MDSINE uses an adaptive *L*_*1*_ or “lasso”-type regularization technique for inferring the distribution over microbial growth, interaction, and perturbation effect parameters in the gLV model. The BAL algorithm is adaptive in the sense that it allows for different degrees of regularization on each coefficient, i.e., each OTU has a different prior amount of interaction parameter shrinkage that is learned from the data. See Additional file 2 for complete details on the BAL model and inference algorithm.

The Bayesian Variable Selection (BVS) algorithm in MDSINE also infers the distribution over microbial growth, interaction, and perturbation effect parameters in the gLV model. However, in this approach we use a variable selection technique that directly models the presence or absence of microbe–microbe interactions or perturbation effects. Our model effectively learns a qualitative interaction network on the OTUs and perturbation effects as well as a quantitative interaction and perturbation effects matrix. Stated another way, the MLCRR and BAL regularization-based approaches in MDSINE assume all edges are present in the network, whereas the BVS approach additionally learns the presence/absence of the edges. The BVS algorithm also allows us to readily calculate Bayes factors [35], which provide a formal method for comparing two alternative models given evidence (data). For MDSINE, we use Bayes factors to assess alternative models indicating the presence or absence of an interaction or perturbation effect given the data. See Additional file 2 for complete details on the BVS model, the inference algorithm, and calculations of Bayes factors from the model.

#### Benchmarking with simulated data

We used the *C. difficile* infection dataset to obtain rough estimates of the scale and variability of growth rates, interaction coefficients, and initial concentrations of microbes. Estimates were obtained by solving the “gradient-matching” linear regression system using the pseudoinverse solution and forcing growth and self-interaction parameters to be positively and negatively valued, respectively. These estimates were then used to randomly sample growth and interaction parameters for a hypothetical 10-OTU system assuming a 20 % probability of interaction between OTU pairs (roughly what we observed in the *C. difficile* infection data).

To obtain dynamical systems models for testing simulations, we required that all OTUs be present at steady state and have coefficients of variation for their trajectories >0.25 for 75 % of initial concentrations tested (evaluated by randomly generating initial conditions numerous times and numerically integrating the gLV equations for each initial condition). The resulting dynamical systems model parameters were then used to generate simulated data for benchmarking. These simulations involved random sampling of initial concentrations of OTUs for ten “subjects” and numerical integration of the gLV equations using the initial concentrations to generate noise-free trajectories. We investigated different time point sampling resolutions, with designs of 27, 18, 12, or 8 time points chosen to mimic real experimental designs for in vivo microbiome time-series datasets. See Additional file 2 for complete details on the data simulations.

Noisy count data and biomass data were then simulated from noise-free trajectories using Dirichlet Multinomial Distribution (DMD) [19, 20] and Normal Distribution models, respectively. The dispersion parameter for the Dirichlet Multinomial distribution was estimated from the *C. difficile* infection experimental data (16S rRNA amplicon sequencing counts) using a maximum likelihood procedure implemented in the MGLM toolkit [36]. Note that the DMD model assumes compositionality of data (counts sum to a fixed constant for each sample), with over-dispersion of counts. MDSINE uses an alternative noise model based on the Negative Binomial Distribution (NBD), which also assumes over-dispersion of counts but does not assume compositionality of data (counts are generated independently for each sample conditional on a shared intensity parameter). Note that we intentionally tested performance of MDSINE on data generated with the DMD, a different noise model than implemented in MDSINE, to evaluate robustness of our method to compositionality of data.

We evaluated simulated results for each of the algorithms implemented in the MDSINE package using the following metrics: (a) the root mean square error (RMSE) of the estimated growth rates compared with ground-truth growth rates; (b) the RMSE of the estimated interaction parameters compared with ground-truth interaction parameters; (c) the area under the receiver operator curve (AUC ROC) for presence/absence of interactions, for inferred interaction networks compared with the ground-truth network; (d) the RMSE of the predicted OTU trajectories compared with the ground-truth trajectories on unseen initial conditions (i.e., training on *n* − 1 subjects and prediction of the held-out subject given initial conditions for that subject). The above metrics were computed on 400 samples for each of the regimens described above. See Additional file 2 for complete details on the evaluation procedure.

#### C. difficile *infection resistance predictions*

In order to predict which bacterial compositions optimally confer resistance against *C. difficile* infection, we began by calculating which of the 2^13^ − 1 possible bacterial combinations were predicted to have biologically meaningful steady-state levels (i.e., concentrations of species ≥0) and were stable (i.e., all the eigenvalues of the Jacobian matrix evaluated in the steady state have negative real part [8]; see Additional file 2 for further details). Steady-state concentrations and stability determinations were made from a subsample of MCMC samples obtained from running the BVS inference algorithm (1125 MCMC samples, using a thinning rate of 20 from the full 22,500 MCMC samples). States with a very high confidence of stability (estimated probability of stability >0.9) were retained. Starting from the predicted concentration profile of each stable MCMC sample, we simulated a *C. difficile* infection challenge of 10^5^ CFU/g, corresponding to a high infectious dose experimentally. We numerically integrated the corresponding gLV system for 28 days and determined for each microbiota state the level of *C. difficile* infection at the end of the numerical experiment as the median concentration across the corresponding MCMC samples. We then determined which states (for 1 through all species in the defined microbiota) led to the minimal median *C. difficile* concentrations at 28 days.

#### Prediction of stable steady state profiles for probiotic cocktail

In order to determine if switching to a dietary regime with low-fiber content would lead to a reduced number of admissible steady states as well as to reduced biodiversity, we estimated which of the 2^13^ − 1 possible bacterial combinations had a stable and biologically meaningful density profile in high- and low-fiber diet conditions. To determine the stability profiles in the presence of the low-fiber perturbations, we calculated the net growth parameter for each strain *i* as the sum of its inferred growth rate *α*_*i*_ and its susceptibility to low-fiber diets *γ*_*i*_ (perturbation effect parameter). We repeated this analysis using 1125 MCMC samples (with a thinning rate of 20 from the full 22,500 MCMC samples) obtained from running the BVS inference algorithm. We then determined the number of predicted stable steady states in each condition and tested the null hypothesis of equal numbers of stably coexisting strains within stable states under both dietary regimens by applying a Wilcoxon rank sum test.

#### Prediction of keystoneness of species

To investigate the importance of specific strains in the Clostridia community from our probiotic dataset, we developed a quantitative measure of “keystoneness” in the MDSINE package. The measure starts from the community composition that allows the largest number of strains to stably coexist, and then removes each strain from the community in turn. Steady-state concentrations are calculated as described in the above subsection. The Euclidean distance between the concentrations of the starting profile and that of each of the profiles with one strain removed is then calculated, with the calculation excluding the contribution of the removed strain. The strains are then ranked based on the magnitude of the distances obtained as a consequence of their removal.

### *C. difficile* infection in gnotobiotic mice

#### Commensal bacterial cultures

We developed a set of defined human commensal bacteria, which we term the GnotoComplex flora, based on extensive review of the literature [37–52]. Strains were chosen to approximate phylogenetic diversity and key roles of the microbiota in the host, including the ability to transform bile acids and degrade a variety of dietary compounds (Additional file 1: Table S2). The GnotoComplex bacteria were also selected so that they are all distinguishable at the V4 region of the 16S rRNA gene (three or more nucleotide differences in the region) to ensure that the strains can all be differentiated with our high-throughput sequencing method. The GnotoComplex bacterial strains were purchased from either the American Type Culture Collection (ATCC) or the Leibniz Institute DSMZ-German Collection of Microorganisms and Cell Cultures (DSMZ) and propagated according to the propagation procedure on the product sheet for each strain (Additional file 1: Table S2). Aliquots were prepared and stored at −80 °C until needed for the preparation of the bacterial inoculum. An aliquot from each bacterial strain was removed from the −80 °C freezer and plated for isolation onto either Tryptic Soy base with 5 % sheep blood agar (TSA) for the aerobic strains or pre-reduced *Brucella* base blood agar containing 5 % sheep blood, hemin, and vitamin K (BMB) for the anaerobic strains. The TSA plates were incubated at 37 °C in ambient air while the BMB plates were incubated at 37 °C in an anaerobic chamber containing an atmosphere of 10 % hydrogen, 10 % carbon dioxide and 80 % nitrogen. The plates were inspected for purity and Gram stains were performed. Brain Heart Infusion broth (BHI; 5 mL; Anaerobe Systems) was inoculated with a single colony of each bacterial strain and incubated overnight under appropriate conditions. The OD600 was determined and the suspensions diluted with BHI if necessary. The bacterial inoculum for mice was prepared by combining the individual bacterial strains based on their OD to ensure that each strain was equally represented.

#### *Clostridium difficile* culture

*C. difficile* strain 43255 was purchased from the ATCC and propagated according to the product sheet. For the preparation of spores, *C. difficile* was plated onto BMB to produce a lawn of growth and incubated in an anaerobic chamber for 10 days. The bacteria were recovered from the plate using a sterile cotton swab that had been pre-moistened with sterile phosphate-buffered saline (PBS). The bacteria were suspended in 15 mL of PBS. The suspension was heated in an 80 °C water bath for 10 min to kill the vegetative cells. Aliquots (1 ml) were prepared, flash frozen using liquid nitrogen, and stored at −80 °C. One aliquot was removed from the −80 °C freezer and serially diluted with PBS and plated onto BHI agar plates containing hemin, vitamin K, and 0.1 % (w/v) of taurocholate. The plates were incubated in an anaerobic chamber for 96 h and the spore concentration determined.

#### Gnotobiotic mouse experiments

Germ-free mice were bred and maintained in isolators in the Massachusetts Host-Microbiome Center at Brigham and Women’s Hospital. Five individually caged, male, 8–10-week-old, germ-free C57BL/6 mice were orally gavaged at day 0 with 200 μl of the GnotoComplex bacterial mixture for a total inoculum of ≈ 10^8^ CFU per mouse. Fecal pellets were collected at 0.75, 1, 2, 3, 4, 6, 8, 10, 14, 17, 21, 24, and 28 days post-inoculation with the GnotoComplex strains and stored at −80 °C in 10 % PBS buffer. After fecal sample collection on day 28, mice were orally gavaged with 5 × 10^3^*C. difficile* spores. Fecal pellets were collected at 0.75, 1, 2, 3, 4, 6, 8, 10, 14, 17, 21, 24, and 28 days post-infection with *C. difficile* and stored at −80 °C in 10 % PBS buffer.

#### 16S rRNA sequencing

Bacterial genomic DNA was extracted using the Mo Bio Power Fecal DNA Isolation kit (Mo Bio Laboratories) according to the manufacturer’s instructions for high yields of DNA. To increase the DNA yields, the following modifications were used. An additional bead beater step using the Faster Prep FP120 (Thermo) at 6 m/s for 1 min was used instead of vortex agitation. Incubation with buffers C2 and C3 was increased to 10 min at 4 °C. Starting nucleic acid concentrations were determined by a Qubit Fluromoter (Life Technologies). A multiplexed amplicon library covering the 16S rDNA gene V4 region was prepared using the protocol of [53] with dual-index barcodes. The aggregated library pool was size selected from 300–500 bp on a pippin prep 1.5 % agarose cassette (Sage Sciences) according to the manufacturer’s instructions. The concentration of the pool was measured by qPCR (Kapa Biosystems) and loaded onto the MiSeq Illumina instrument (300 bp kit) at 6–9 pM with 40 % phiX spike-in to compensate for low base diversity according to Illumina’s standard loading protocol. Sequences were deposited in the NIH Sequence Read Archive (SRA accession SRP065075).

#### Bioinformatic processing of sequences

Raw sequencing reads were processed using custom Python scripts, which perform denoising, quality filtering, and alignment against the 16S rRNA gene sequences of the species in the defined community. To reduce alignment error, low quality bases (Q < 35) were trimmed using a sliding window (window size = 50 nucleotides). Reads with ambiguous characters or shorter than 250 nucleotides after trimming were removed. Finally, trimmed reads were aligned against a custom database of 16S rRNA sequences using blastn (ncbi-blast-2.2.29+) with default parameters. The blastn database was built using full-length 16S rRNA sequences of the GnotoComplex strains, which were extracted from genome reference sequences in National Center for Biotechnology Information (NCBI; http://www.ncbi.nlm.nih.gov/) or downloaded nucleotide sequences from NCBI for cases in which reference sequences were not available. After the alignment, sequencing reads were assigned to species using the criteria: best hit with identity ≥99 %, alignment length ≥250 nucleotides, no gaps, mismatches ≤2 nucleotides, and all bases aligned within the V4 hypervariable of the database sequences. To identify the V4 hypervariable region for the species in the defined community, the full-length 16S rRNA sequences were aligned against the Silva database [54] of the V4 region using nhmmer [55].

### Quantification of bacterial biomass via qPCR

The universal 16S rRNA qPCR primer (16S-F1048, GTG STG CAY GGY TGT CGT CA; 16S-R1175, ACG TCR TCC MCA CCT TCC TC) was used. The reaction mixture for the SYBR Green assay contained 2U SYBR Green PCR Master Mix (PE Applied Biosystems), 20 pmol of forward and reverse primer and 8 μl of 1:100 diluted extracted DNA. The qPCRs were run on a Bio Rad CFX96 Real time PCR system under standard thermal-cycling conditions, consisting of an initial 10 min of denaturation at 95 °C, followed by 39 cycles of 15 s of denaturation at 95 °C and 60 °C for 60 s of annealing/extension. To quantify bacterial biomass, standard curves were prepared using genomic DNA purified from germ-free mouse stool spiked with serial tenfold dilutions of quantified *Escherichia coli*.

### Probiotic stability experiments in gnotobiotic mice

#### Bacterial cultures and gnotobiotic experiments

Germ-free IQI mice were purchased from Sankyo Laboratories, Japan and maintained in germ-free vinyl isolators in the animal facility of RIKEN. Thirteen Treg-inducing Clostridia strains (strains 1, 3, 8, 18 were omitted from the previously reported VE202 cocktail consisting of 17 Treg-inducing strains [24]) were individually cultured in modified Eggerth Gagnon (EG) broth under strictly anaerobic conditions (80 % N_2_, 10 % H_2_, 10 % CO_2_) at 37 °C in an anaerobic chamber (Coy Laboratory Products) to confluence. The cultured bacterial strains were then mixed at equal amounts of media volume and the mixture of 13 strains were orally inoculated into seven IQI germ-free adult mice. Five of the mice were fed with a gamma ray-sterilized high-fiber diet (CMF chow, Oriental Yeast Japan) for 5 weeks, then switched to a low-fiber diet (AIN93G-fomula diet, Oriental Yeast Japan) for 2 weeks, and returned to and maintained on the CMF high-fiber diet for 2 weeks. Two of the mice were maintained on the high-fiber diet for 5 weeks and were not switched to the low-fiber diet. Fecal samples were collected daily or every other day. Fecal pellets were collected at days 1–21 (daily), 23, 25, 27, 29, 31, 33, 35–60 (daily), 62, 63, and 65 for the five mice receiving the dietary perturbation and at days 1–21 (daily), 23, 25, 27, and 29 for the two mice not receiving the perturbation.

#### Quantification of bacterial concentrations via qPCR

Bacterial genomic DNA was extracted from 1–2 fecal pellets using QIAamp DNA Stool Mini Kit (Qiagen). The amount of DNA was quantified using a Qubit dsDNA HS assay kit and Qubit fluorometer (Invitrogen). DNA was then subjected to qPCR using Thunderbird SYBR qPCR Mix (TOYOBO) and a LightCycler 480 (Roche) with primers specific to 16S rRNA genes of the 13 Clostridia strains (see Additional file 1: Table S4). Quantification of each strain in each sample was accomplished using standard curves of known concentrations of DNAs purified from each strain individually cultured in vitro. Strain densities in each sample were calculated by dividing the above absolute quantification numbers by the weight of the extracted fecal DNA.

## Additional files

Additional file 1: Tables S1–4. Supplemental tables. (DOCX 21 kb) Additional file 2: Supplemental methods. (PDF 372 kb) Additional file 3: Figure S1. Performance of the MDSINE inference algorithms on simulated data with different sequencing depths. Simulations assumed an underlying dynamical systems model with ten species observed over 30 days with 27 time points sampled and an invading species at day 10. Performance of the four MDSINE inference algorithms, maximum likelihood ridge regression (MLRR), maximum likelihood constrained ridge regression (MLCRR), Bayesian adaptive lasso (BAL), and Bayesian variable selection (BVS), were compared. Algorithm performance was assessed using four different metrics: (**a**) root mean-square error (RMSE) for microbial growth rates; (**b**) RMSE for microbial interaction parameters; (**c**) RMSE for prediction of microbe trajectories on held-out subjects given only initial microbe concentrations for the held-out subject; and (**d**) area under the receiver operator curve (AUC ROC) for the underlying microbial interaction network. Lower RMSE values indicate superior performance, whereas higher AUC ROC values indicate superior performance. (PDF 182 kb) Additional file 4: Figure S2. Experimental design for *Clostridium difficile* infection studies in gnotobiotic mice. Five adult germfree mice were gavaged with 23 human commensal bacterial type strains, chosen to capitulate the phylogenetic diversity and key physiologic capabilities of a native gut flora (see “Methods” for details). After allowing 28 days for the commensal flora to establish, mice were gavaged with *C. difficile* spores and monitored for an additional 28 days. Fecal pellets were collected at days 0.75, 1, 2, 3, 4, 6, 8, 10, 14, 17, 21, 24, and 28 of the initial colonization and at days 0.75, 1, 2, 3, 4, 6, 8, 10, 14, 17, 21, 24, and 28 post-infection with *C. difficile*. Fecal samples were interrogated via high-throughput 16S rRNA sequencing to determine abundances of species and 16S rRNA qPCR using universal primers to estimate the total bacterial biomass present. After bioinformatics processing (see “Methods”), 13 strains were found to be consistently detectable in pre-infection stool samples. (PDF 87 kb) Additional file 5: Figure S3. Experimental design for probiotic stability studies in gnotobiotic mice. Seven adult germ-free mice were gavaged with 13 Clostridia strains from the VE202 probiotic cocktail [24]. Five mice were maintained on a standard high-fiber diet for 5 weeks, after which mice were switched to a low-fiber diet for 2 weeks and then switched back to the high-fiber diet for another 2 weeks; an additional two mice were inoculated with the same strains but were not subjected to the low-fiber dietary perturbation. Fecal pellets were collected at days 1–21 (daily), 23, 25, 27, 29, 31, 33, 35–60 (daily), 62, 63, and 65 for the five mice receiving the low-fiber dietary perturbation and at days 1–21 (daily), 23, 25, 27, and 29 for the two mice not receiving the perturbation. (PDF 42 kb) Additional file 6: Figure S4. Forecasts of microbial concentration trajectories for the gnotobiotic mice probiotic stability experiments. The forecasts were obtained using a hold-one-subject-out procedure. Briefly, MDSINE was run on all data from all but one of the mice (the held-out subject) and model parameters were inferred. Using the inferred model parameters (including for the perturbation) and the measured concentrations of the microbiota at an initial time point for the held-out mouse, the trajectories of the microbiota for the held-out mouse were then forecast for all the remaining time points; the procedure was repeated for each mouse in turn. Solid lines denote predicted trajectories and symbols denote actual data. (PDF 7552 kb) Additional file 7: Figure S5. Predicted stability and steady state concentrations (log_10_ ng strain DNA/μg total fecal DNA) for all combinations of the 13 Clostridia strains in mice fed either high-fiber (standard) or low-fiber diets in the probiotic stability experiment. *Columns* and *rows* were ordered using hierarchical clustering using Euclidean distance with Ward linkage. No significant differences were found in the predicted stable biodiversity profiles between the high-fiber and low-fiber dietary regimes (number of strains across all predicted stable states was not significantly different; Wilcoxon rank sum test *p* value = 0.096). (PDF 4845 kb) Additional file 8: Figure S6. Directed microbe–microbe and microbe–perturbation effect network for the gnotobiotic mice probiotic stability experiment. Edge thickness denotes the magnitude of the evidence favoring presence of the interaction (Bayes factor). Only edges with strong evidence (Bayes factor ≥10) are displayed. (PDF 230 kb)
