# Supplementary material for: MDSINE: Microbial Dynamical Systems INference Engine for microbiome time-series analyses
Source: Genome Biol. 2016 Jun 3;17:121. doi: 10.1186/s13059-016-0980-6 (PMC4893271; doi:10.1186/s13059-016-0980-6)
Supplement: Additional file 1: Tables S1–4. — Supplemental tables. (DOCX 21 kb) [file 13059_2016_980_MOESM1_ESM.docx]

**Tables**

| **Algorithm** | **How estimates trajectories and gradients?** | **Constrains growth and self-interaction parameters?** | **How avoids over-fitting?** | **Advantages** |
| --- | --- | --- | --- | --- |
| MLRR  (maximum-likelihood ridge regression) | Trajectories: uses data directly  Gradients: first-order difference-based method | N | L_2_-regularization | Fastest |
| MLCRR  (maximum-likelihood constrained ridge regression) | Same as MLRR | Y | L_2_-regularization | Relatively fast |
| BAL  (Bayesian adaptive lasso) | Bayesian Adaptive Penalized Counts Splines algorithm to estimate continuous-time trajectories and gradients using Negative Binomial Distribution-based error model | Y | Regularization with Bayesian lasso (L_1_-penalty) | Relatively fast, estimates variability in inferences and predictions |
| BVS  (Bayesian variable selection) | Same as BAL | Y | Variable selection (determines presence/absence of interactions) | Estimates variability in inferences and predictions, calculates Bayes Factors useful for determining which interactions best supported by the data |

**Supplemental Table 1**: Summary of the dynamical systems inference algorithms implemented in MDSINE.

| **Organism** | **Strain ID** | **Previously described functions** | **Relative abundance at 28 days** | | | | |
| --- | --- | --- | --- | --- | --- | --- | --- |
|  |  |  | M1 | M2 | M3 | M4 | M5 |
| *Akkermansia muciniphila* | ATCC BAA-835 | l | 31.21% | 32.85% | 35.36% | 35.13% | 30.24% |
| *Bacteroides fragilis* | ATCC 25825 | a,c,d,l,m,n,q | 11.27% | 9.09% | 7.96% | 6.28% | 11.11% |
| *Bacteroides ovatus* | ATCC 8483 | p | 32.52% | 26.79% | 25.56% | 29.50% | 26.99% |
| *Bacteroides vulgatus* | ATCC 8482 | a,b,c,l,m,n,q | 8.09% | 6.53% | 8.24% | 6.39% | 7.77% |
| *Clostridium hiranonis* | DMS-13275 | f | 0.10% | 0.11% | 0.03% | 0.10% | 0.09% |
| *Clostridium ramosum* | DSM 1402 | a,h,i,o | 2.10% | 6.57% | 7.71% | 4.07% | 8.76% |
| *Clostridium scindens* | DSM 5676 | f | 0.14% | 0.32% | 0.39% | 0.25% | 0.18% |
| *Enterococcus faecalis* | ATCC 29200 | e,l | 0.00% | 0.00% | 0.01% | 0.00% | 0.00% |
| *Eshchericia coli* | Nissle 1917 | c,d,q | 4.50% | 6.15% | 1.62% | 5.03% | 2.54% |
| *Klebsiella oxytoca* | ATCC 700324 |  | 2.31% | 2.34% | 2.18% | 2.30% | 2.38% |
| *Lactobacillus fermentum* | ATCC 23271 | e,j,l,q | 0.00% | 0.00% | 0.00% | 0.00% | 0.00% |
| *Lactobacillus reuteri* | ATCC 23272 | j,u,p | 0.00% | 0.00% | 0.00% | 0.00% | 0.00% |
| *Parabacteroides distasonis* | ATCC 8503 | a,k,l,m,n | 2.39% | 3.52% | 3.35% | 3.95% | 3.42% |
| *Prevotella melaninogenica* | ATCC 25845 |  | 0.00% | 0.00% | 0.00% | 0.00% | 0.00% |
| *Propionibacterium acnes* | ATCC 11827 | a,m | 0.00% | 0.00% | 0.00% | 0.00% | 0.00% |
| *Proteus mirabilis* | ATCC 29906 |  | 1.40% | 0.99% | 1.88% | 1.02% | 1.38% |
| *Roseburia hominis* | DMS 16839 | c,g | 2.24% | 2.07% | 3.08% | 4.41% | 2.65% |
| *Ruminococcus obeum* | ATCC 29174 |  | 1.72% | 2.67% | 2.66% | 1.56% | 2.48% |
| *Staphylococcus epidermidis* | ATCC 12228 |  | 0.00% | 0.00% | 0.00% | 0.00% | 0.00% |
| *Streptococcus mitis* | ATCC 49456 |  | 0.00% | 0.00% | 0.00% | 0.00% | 0.00% |

**Supplemental Table 2**: Bacterial strains used in the GnotoComplex human commensal microbiota mixture. Strains were selected based on extensive literature review (see Methods). Previously described activities of the strains include: (a) acetate, (b) α-glucuronidase, (c) β-glucuronidase, (d) bile acid dehydrogenase, (e) bile acid dehydrolase, (f) bile acid hydroxylase, (g) butyrate, (h) formate, (i) H_2_, (j) lactate, (k) CH_4_, (l) mucin degradation, (m) propionate, (n) succinate, (o) urobilogen, (p) vitamin B12, (q) vitamin K. Percentages indicate relative abundances of organisms at 28 days post-inoculation prior to infection with *C. difficile*, based on the 16S rRNA MiSeq data. M1-M5 = mouse 1-5.

|  | **MLRR** | **MLCRR** | **BAL** | **BVS** |
| --- | --- | --- | --- | --- |
| *C. difficile* infection | 1.32 | 1.16 | 0.56 | 0.66 |
| Probiotic stability | 0.24 | 0.24 | 0.25 | 0.26 |

**Supplemental Table 3**: Performance of the new MDSINE inference algorithms and our previous method on the task of predicting microbial growth concentrations over time. Predictive performance of MDSINE was assessed using a hold-one-out cross-validation procedure. In this procedure, MDSINE was run on all data from all but one of the mice in the experiment (the held-out subject) and model parameters were inferred. Using the inferred model parameters (including for the perturbation in the case of the probiotic stability data) and the measured concentrations of the microbiota at an initial time-point for the held-out mouse, the microbial concentration trajectories for that mouse were then forecast for all the remaining time-points. This procedure was repeated for each mouse in turn, and predictive performance was evaluated as the root-mean squared error (RMSE) between the predicted trajectory and the actual data. Lower RMSE values indicate superior performance. Units of RMSE are CFU/g for the *C. difficile* data and ng strain DNA / μg total fecal DNA for the probiotic data. MLRR = maximum-likelihood ridge regression, MLCRR = maximum-likelihood constrained ridge regression, BAL = Bayesian adaptive lasso, BVS = Bayesian variable selection. For the *C. difficile* infection experiments, the Bayesian algorithms significantly outperformed the maximum-likelihood approach (*p*-values of 0.0079 for MLCRR vs. BAL, 0.0317 for MLCRR vs. BVS, using Wilcoxon ranksum test) with no significant differences between the two Bayesian algorithms (*p*-value = 0.310). For the probiotic stability experiments, we found no significant differences in performance among the algorithms evaluated (*p*-values of 0.841 for MLCRR vs BAL, 0.310 for MLCRR vs BVS, and 0.421 for BAL vs BVS).

| **ID** | **Forward** | **Reverse** |
| --- | --- | --- |
| VE202 st.4-specific | 5'-CGAGCGAAGCGGTTTCA-3' | 5'-TTCTAACTGTTATCCCCCAGTGTA-3' |
| VE202 st.6-specific | 5'-GAGCGAAGCAGTAAGACG-3' | 5'-CTAACTGTTATCCCCCTGTATGA-3' |
| VE202 st.7-specific | 5'-CGGCGTGCCTAACACAT-3' | 5'-GTCCGCCACTCAGTCAATCA-3' |
| VE202 st.9-specific | 5'-TGGGGAACCTGCCCTATACA-3' | 5'-CGGAGCTTTTCACACCGAAT-3' |
| VE202 st.13-specific | 5'-ACGGAGCTTACGTTTTGAA-3' | 5'-GGCTGTTATCCCCCTCTGA-3' |
| VE202 st.14-specific | 5'-GCGCTGTTTTCAGAATCTT-3' | 5'-ACCGGAGTTTTTCACACTAC-3' |
| VE202 st.15-specific | 5'-CGGCGTGCCTAACACAT-3' | 5'-CGCCACTCAGTCATCTCAGAA-3' |
| VE202 st.16-specific | 5'-AGTCGAACGAAGCGATTTAAC-3' | 5'-CCGGAGTTTTTCACACTGTAT-3' |
| VE202 st.21-specific | 5'-GCGCTTTACTTAGATTTCTTCG-3' | 5'-CCATGCGGTACTGTGGT-3' |
| VE202 st.26-specific | 5'-GGAGATGAAGGCGGCT-3' | 5'-ACCCTCTCAGGTCGGC-3' |
| VE202 st.27-specific | 5'-GCAGTCGAACGGAGTTATG-3' | 5'-CACACTGCCTCATGTGAAG-3' |
| VE202 st.28-specific | 5'-CAGTCGAACGAAGCATCTTATAG-3' | 5'-GATCCATCTCACACCACCT-3' |
| VE202 st.29-specific | 5'-GGTGTAGGTGGGTATGGAC-3' | 5'-AAATCCTCTTTACAGGAGCG-3' |

**Supplemental Table 4**: Primers used for measurement of the concentrations of Clostridial strains in the gnotobiotic mice probiotic stability experiments.
