# Supplementary material for: MDSINE: Microbial Dynamical Systems INference Engine for microbiome time-series analyses
Source: Genome Biol. 2016 Jun 3;17:121. doi: 10.1186/s13059-016-0980-6 (PMC4893271; doi:10.1186/s13059-016-0980-6)
Supplement: Additional file 2: — Supplemental methods. (PDF 372 kb) [file 13059_2016_980_MOESM2_ESM.pdf]

Supplemental Information for: Dynamical systems analysis for microbiome time-series studies

Contents

1 Overview of The Microbial Dynamical Systems INference Engine (MDSINE) 2

2 Maximum-likelihood constrained ridge regression (MLCRR) algorithm 2
2.1 Approximation of concentrations and gradients 3
2.1.1 ℓ_2-regularized solution with inequality constraints on parameters 4
2.1.2 Setting of regularization parameters 5

3 Bayesian algorithms 5
3.1 Estimation of OTU trajectories and gradients: Bayesian Adaptive Penalized Counts Splines (BAPCS) 6
3.1.1 Model description 6
3.1.2 Model inference 8
3.1.3 Setting of hyperparameters and initializations 8
3.1.4 Computing trajectory and gradient estimates from samples 8
3.1.5 Scaling factors 9
3.2 Bayesian gLV parameter estimation method 1: Bayesian adaptive lasso (BAL) 9
3.2.1 Model definition 9
3.2.2 Model inference 10
3.2.3 Setting of hyperparameters and initializations 10
3.3 Bayesian gLV parameter estimation method 2: Bayesian variable selection (BVS) 11
3.3.1 Model definition 11
3.3.2 Model inference 12
3.3.3 Bayes factors 13
3.3.4 Setting of hyperparameters and initializations 13

4 Steady-state and stability estimation 14

5 Benchmarking with simulated data 14
5.1 Data simulation procedure 14
5.2 Evaluation metrics 15

1 Overview of The Microbial Dynamical Systems INference Engine (MDSINE)

The Microbial Dynamical Systems INference Engine (MDSINE) software package implements algorithms for inferring dynamical systems models of the microbiota from high-throughput time-series data. For the underlying dynamical systems model, we use the extended generalized Lotka-Volterra (gLV) equations as previously described [1]. For the gLV model for L OTUs measured in S subjects, the rate of change of the concentration of OTU l in subject s is expressed as:

$$\frac{\mathrm{d}f_{ls}}{\mathrm{d}t} = \alpha_l f_{ls}(t) + \sum_{j=1}^{L} \beta_{lj} f_{ls}(t) f_{js}(t) + \sum_{p=1}^{P} \gamma_{lp} f_{ls}(t) u_p(t) \tag{1}$$

The $\boldsymbol{\alpha}$ parameters represent unbounded growth rates, the $\boldsymbol{\beta}$ parameters represent pair-wise microbe-microbe interactions, and the $\boldsymbol{\gamma}$ parameters represent effects of P perturbations. The functions $u_p(t)$ are binary-valued, indicating if the given perturbation is present at time t. Unlike in our previous approach [1], which did not assume constraints on the parameters, here we assume positive growth rates and negative self-interaction rates, i.e., $\alpha > 0$ and $\beta_{ll} < 0$.

We use a "gradient matching" approach to estimate the ODE parameters:

$$\boldsymbol{\theta}_l = [\alpha_l \; \beta_{l1} \cdots \beta_{lL} \; \gamma_{l1} \cdots \gamma_{lP}]^T$$

The concept underlying this approach is that if estimates of the gradient and trajectory values are available, parameters can be estimated by solution of systems of equations rather than systems of differential equations. In the case of the gLV model, the "gradient matching" system of equations is linear, and can be written as:

$$\frac{\mathrm{d}f_{ls}}{\mathrm{d}t} \approx \widehat{f}'_{lst} \approx \alpha_l \widehat{f}_{lst} + \sum_{j=1}^{L} \beta_{lj} \widehat{f}_{lst} \widehat{f}_{jst} + \sum_{p=1}^{P} \gamma_{lp} \widehat{f}_{lst} u_p(t) \tag{2}$$

Here, $\widehat{f}_{lst}$ represents an estimate of the concentration of OTU l in subject s at time-point t, and $\widehat{f}'_{lst}$ represents the corresponding gradient estimate. These estimates are derived from data as described in the sections below. We assume we have measurements of counts Y_{lst} (i.e., obtained via 16S rRNA sequencing) for each OTU l in subject s at time-point t, where there are L OTUs, S subjects and N_s time-points per subject[1]. We also assume we have measurements of total bacterial biomass W_{st} (i.e., obtained via qPCR) for each time-point t in each subject s.

The reduction of the gLV differential equations to a linear system of equations via the "gradient matching" approach enables application of statistical models for linear regression. However, we are still faced with estimating $L^2 + L + LP$ parameters, which will result in an under-determined system for typical datasets. We thus developed several algorithms that use regularization or variable selection techniques during the parameter inference process.

2 Maximum-likelihood constrained ridge regression (MLCRR) algorithm

The overall objective of the MDSINE MLCRR algorithm is to infer point-estimates of growth, self-interaction and interaction parameters for the gLV model from high-throughput 16S rRNA gene sequencing data and qPCR measurements of microbial biomass. We have previously described a maximum likelihood approach, which does not constrain the growth rates or self-interaction parameters. In the present work, we extend our method to constrain growth rates to positive values and self-interaction parameters to negative values. For completeness, we describe both the original algorithm [1] and new algorithm with constrained parameters.

[1] For ease of notation in subsequent sections, we drop the subject-specific subscript on the number of time-points.

2.1 Approximation of concentrations and gradients

We approximate the concentration for OTU l in subject s at time-point t as:

$$\widehat{f}_{lst} = \frac{Y_{lst}}{\sum_l Y_{lst}} W_{st} \tag{3}$$

That is, we approximate the OTU concentration as the relative abundance estimated from the 16S rRNA counts data times the total bacterial biomass measurement.

To estimate gradients of OTU concentrations, we use a discrete approximation method as previously described [1]. Briefly, Equation 1 can be rewritten as:

$$\frac{\mathrm{d}}{\mathrm{d}t} \ln\left(f_{ls}(t)\right) = \alpha_l + \sum_{j=1}^{L} \beta_{lj} f_{js}(t) + \sum_{p=1}^{P} \gamma_{lp} u_p(t). \tag{4}$$

Here, $\frac{\mathrm{d}}{\mathrm{d}t} \ln\left(f_{ls}(t)\right) = \frac{f'_{ls}(t)}{f_{ls}(t)}$ for $f_{ls} \neq 0$. For estimated values $\widehat{f}_{lst}$ at N discrete time points, the time derivative in Equation 4 can be approximated by the forward difference quotient:

$$\left.\frac{\mathrm{d}}{\mathrm{d}t} \ln\left(f_{ls}(t)\right)\right|_{t=t_k} \cong \frac{\widehat{f}'_{lst_k}}{\widehat{f}_{lst_k}} = \frac{\ln\left(\widehat{f}_{lst_{k+1}}\right) - \ln\left(\widehat{f}_{lst_k}\right)}{t_{k+1} - t_k}$$

Using these approximations, the "gradient matching" form of the gLV equations is then:

$$\frac{\widehat{f}'_{lst_k}}{\widehat{f}_{lst_k}} = \frac{\ln\left(\widehat{f}_{lst_{k+1}}\right) - \ln\left(\widehat{f}_{lst_k}\right)}{t_{k+1} - t_k} \approx \alpha_l + \sum_{j=1}^{L} \beta_{lj} \widehat{f}_{jst_k} + \sum_{p}^{P} \gamma_{lp} u_p(t_k). \tag{5}$$

This represents a linear system of equations that can be notated in standard linear regression form. We denote the $(L+1+P) \times L$ dimensional combined parameter matrix as $\Theta = \left[\boldsymbol{\theta}_1, \ldots, \boldsymbol{\theta}_L\right]$.

The dependent variable matrix is denoted:

$$\widehat{F}'_s = \left(\frac{\ln\left(\widehat{f}_{lst_{k+1}}\right) - \ln\left(\widehat{f}_{lst_k}\right)}{t_{k+1} - t_k}\right)_{k,l} \in \mathbb{R}^{(N-1)\times L} \tag{6}$$

The combined $(N-1)S \times L$ dimensional dependent variable matrix for all subjects is then denoted $\widehat{F}' = \begin{bmatrix} \widehat{F}'_1 \\ \vdots \\ \widehat{F}'_S \end{bmatrix}$.

We denote the $(N-1)S \times (L+1+P)$ dimensional design matrix as:

$$X = \begin{bmatrix}
1 & \widehat{f}_{111} & \cdots & \widehat{f}_{1L1} & u_1(t_1) & \cdots & u_P(t_1) \\
1 & \widehat{f}_{112} & \cdots & \widehat{f}_{1L2} & u_1(t_2) & \cdots & u_P(t_2) \\
\vdots & \vdots & & \vdots & \vdots & & \vdots \\
1 & \widehat{f}_{11(N-1)} & \cdots & \widehat{f}_{1L(N-1)} & u_1(t_{N-1}) & \cdots & u_P(t_{N-1}) \\
1 & \widehat{f}_{211} & \cdots & \widehat{f}_{2L1} & u_1(t_1) & \cdots & u_P(t_1) \\
1 & \widehat{f}_{212} & \cdots & \widehat{f}_{2L2} & u_1(t_2) & \cdots & u_P(t_2) \\
\vdots & \vdots & & \vdots & \vdots & & \vdots \\
1 & \widehat{f}_{21(N-1)} & \cdots & \widehat{f}_{2L(N-1)} & u_1(t_{N-1}) & \cdots & u_P(t_{N-1}) \\
\vdots & \vdots & & \vdots & \vdots & & \vdots \\
1 & \widehat{f}_{S11} & \cdots & \widehat{f}_{SL1} & u_1(t_1) & \cdots & u_P(t_1) \\
1 & \widehat{f}_{S12} & \cdots & \widehat{f}_{SL2} & u_1(t_2) & \cdots & u_P(t_2) \\
\vdots & \vdots & & \vdots & \vdots & & \vdots \\
1 & \widehat{f}_{S1(N-1)} & \cdots & \widehat{f}_{SL(N-1)} & u_1(t_{N-1}) & \cdots & u_P(t_{N-1})
\end{bmatrix} \tag{7}$$

The ℓ_2-regularized solution to the linear system is thus expressed as:

$$\Theta_{\boldsymbol{\lambda}} = \arg\min_{\Theta} \left\{ \left\| \widehat{F}' - X\Theta \right\|_F^2 + \left\| \sqrt{D_{\boldsymbol{\lambda}}}\Theta \right\|_F^2 \right\}, \tag{8}$$

where $||.||_F$ indicates the Frobenius norm. Here, $D_{\boldsymbol{\lambda}}$ is a $L+1+P$ diagonal matrix encoding the $\boldsymbol{\lambda}$ penalization parameters corresponding to the dimensions of the subparameters and $\sqrt{D_{\boldsymbol{\lambda}}}$ its principal square root. We place different penalization parameters on the growth, interaction and perturbation effect parameters, and obtain the diagonal matrix $D_{\boldsymbol{\lambda}} \in \mathbb{R}^{L+1+P \times L+1+P}$ with entries:

$$D_{\boldsymbol{\lambda}} = \begin{bmatrix} \lambda_\alpha & 0 & 0 & \cdots & \cdots & 0 \\ 0 & \lambda_\beta & 0 & \cdots & \cdots & 0 \\ 0 & 0 & \lambda_\beta & \cdots & \cdots & 0 \\ \vdots & \vdots & \vdots & \ddots & \vdots & \vdots \\ 0 & 0 & 0 & \cdots & \lambda_\gamma & 0 \\ 0 & 0 & 0 & \cdots & 0 & \lambda_\gamma \end{bmatrix}. \tag{9}$$

The analytic solution to Equation 8 without constraints on the parameters Θ is given by (see our previous work [1] for details):

$$\Theta_{\boldsymbol{\lambda}} = \left(X^T X + D_{\boldsymbol{\lambda}}\right)^{-1} X^T \widehat{F}'. \tag{10}$$

2.1.1 ℓ_2-regularized solution with inequality constraints on parameters

We solve Equation 8 subject to constraints on the parameters Θ using a Quadratic Programming (QP) approach. The general QP problem can be stated as:

$$\mathbf{a}^* = \arg\min_{\mathbf{a}} \left\{ \frac{1}{2}\mathbf{a}^T H \mathbf{a} + \mathbf{c}^T \mathbf{a} \right\} \tag{11}$$

with matrix H and vector $\mathbf{c}$. Casting Equation 8 as a QP problem gives us:

$$\begin{aligned} \left\| X\Theta - \widehat{F}' \right\|_2^2 + \left\| \sqrt{D_{\boldsymbol{\lambda}}}\Theta \right\|_2^2 &= \left\| \tilde{X}\,\mathrm{vec}(\Theta) - \mathrm{vec}(\widehat{F}') \right\|_2^2 + \left\| \sqrt{\tilde{D}_{\boldsymbol{\lambda}}}\,\mathrm{vec}(\Theta) \right\|_2^2 && (12) \\ &= \left(\tilde{X}\,\mathrm{vec}(\Theta) - \mathrm{vec}(\widehat{F}')\right)^T \left(\tilde{X}\,\mathrm{vec}(\Theta) - \mathrm{vec}(\widehat{F}')\right) + \mathrm{vec}(\Theta)^T \tilde{D}_{\boldsymbol{\lambda}}\,\mathrm{vec}(\Theta) \\ &= \mathrm{vec}(\Theta)^T \left(\tilde{X}^T\tilde{X} + \tilde{D}_{\boldsymbol{\lambda}}\right) \mathrm{vec}\left(\Theta^T\right) - 2\,\mathrm{vec}(\widehat{F}')^T \tilde{X}\,\mathrm{vec}\left(\Theta^T\right) + \|\widehat{F}'\|_2^2 && (13) \end{aligned}$$

$$\text{where} \quad \tilde{D}_{\boldsymbol{\lambda}} = \begin{pmatrix} D_{\boldsymbol{\lambda}} & 0 & \dots & 0 \\ 0 & D_{\boldsymbol{\lambda}} & \dots & 0 \\ \vdots & \vdots & \ddots & \vdots \\ 0 & 0 & \dots & D_{\boldsymbol{\lambda}} \end{pmatrix} \in \mathbb{R}^{L(L+1+P)\times L(L+1+P)}$$

$$\text{and} \quad \tilde{X} = \begin{pmatrix} X & 0 & \dots & 0 \\ 0 & X & \dots & 0 \\ \vdots & \vdots & \ddots & \vdots \\ 0 & 0 & \dots & X \end{pmatrix} \in \mathbb{R}^{L(N-1)S\times L(L+1+P)}$$

and vec outputs the (columnwise) vectorization of a matrix. Translating Equation 12 into the general QP form as in Equation 11 yields:

$$\mathbf{a}^* = \arg\min_{\mathbf{a}} \left\{ \frac{1}{2}\mathbf{a}^T H \mathbf{a} + \mathbf{c}^T \mathbf{a} \right\} \quad \text{with} \quad \begin{cases} \mathbf{a} = \text{vec}\,(\Theta) \in \mathbb{R}^{L(L+1+P)\times 1} \\ H = 2(\tilde{X}^T\tilde{X} + \tilde{D}_{\boldsymbol{\lambda}}) \in \mathbb{R}^{L(L+1+P)\times L(L+1+P)} \\ \mathbf{c} = -2\tilde{X}^T\,\text{vec}\big(\widehat{F}'\big) \in \mathbb{R}^{L(L+1+P)\times 1}. \end{cases} \tag{14}$$

To initialize the QP algorithm, we use the solution for unconstrained parameters given in Equation 10. We then solve Equation 14 specifying the following criteria for the lower and upper bounds $\mathbf{a_{lb}}$ and $\mathbf{a_{ub}}$ on $\mathbf{a}$:

$$\mathbf{a_{lb}} = \big[\underbrace{\underbrace{0, -\infty, \cdots, -\infty}_{L+P+1}, \underbrace{0, -\infty, \cdots, -\infty}_{L+P+1}, \cdots}_{L\text{ times}}\big]$$

$$\mathbf{a_{ub}} = \big[\infty, \underbrace{\underbrace{0, \infty, \cdots, 0}_{L+P+i}, \cdots}_{i=2,\cdots,L}\big].$$

2.1.2 Setting of regularization parameters

We set the regularization parameters $\boldsymbol{\lambda}$ using k-fold cross validation as in our prior work [1]. Briefly, our algorithm iterates across a three-dimensional mesh to determine the setting $\boldsymbol{\lambda}^*$ such that the mean-squared stepwise prediction error (or residual) on held-out data is minimized, $\text{res}(\boldsymbol{\lambda}) = \sum_{i=1}^{\binom{S}{k}} \frac{1}{L\cdot(N-1)^{\text{test},i}} \|\widehat{F}'^{\,\text{test},i} - X^{\text{test},i}\Theta_{\boldsymbol{\lambda}}^{\text{train},i}\|_2^2$. This procedure additionally yields the corresponding $\Theta_{\boldsymbol{\lambda}^*}$ parameters for the optimal $\boldsymbol{\lambda}^*$ setting, which is used for numerical predictions from new sets of initial conditions and the perturbations profiles.

3 Bayesian algorithms

The overall objective of the MDSINE Bayesian methods is to infer distributions of growth, self-interaction and interaction parameters for the gLV model from high-throughput 16S rRNA gene sequencing data and measurements of microbial biomass. Inferred distributions of parameters are then used to compute summary measures, including median predicted trajectories for OTUs, as well as measures of variability and strength of model selection evidence. As with the MLCRR maximum likelihood-based method, we use a "gradient matching" approach to infer parameters for the gLV nonlinear differential equations.

For the Bayesian methods, we use a two-step estimation procedure. First, we estimate trajectories and gradients of OTU concentrations directly from sequencing counts and biomass data. Then, we use the estimated trajectories and gradients to infer the gLV parameters using Bayesian linear modeling techniques. For both steps, we use custom Markov Chain Monte Carlo (MCMC) algorithms.

We define some general notation shared by the Bayesian models[2]. We denote the dependent variables in the gLV "gradient matching" Equation 2 by $\widehat{F}'_l$, and the SN-dimensional concatenated vector of gradients for OTU l in all subjects and at all measured time-points as:

$$\widehat{F}'_l = \left[\widehat{f}'_{l11}\ \widehat{f}'_{l12}\ \cdots\ \widehat{f}'_{l1N}\ \widehat{f}'_{l21}\ \cdots\ \widehat{f}'_{l2N}\ \cdots\ \widehat{f}'_{lS1}\ \cdots\ \widehat{f}'_{lSN}\right]^T \tag{15}$$

We denote the design matrix for gLV "gradient matching" Equation 2 as:

[2]Note that the corresponding matrices are defined somewhat differently for the maximum-likelihood approach, both for ease of notation and also because the OTU concentration gradients are approximated differently.

$$
X_l = \begin{bmatrix}
\widehat{f}_{l11} & \widehat{f}_{l11}\widehat{f}_{111} & \cdots & \widehat{f}_{l11}\widehat{f}_{l11}\widehat{f}_{L11} & \widehat{f}_{l11}u_1(t_1) & \cdots & \widehat{f}_{l11}u_P(t_1) \\
\widehat{f}_{l12} & \widehat{f}_{l12}\widehat{f}_{112} & \cdots & \widehat{f}_{l12}\widehat{f}_{L12} & \widehat{f}_{l12}u_1(t_2) & \cdots & \widehat{f}_{l12}u_P(t_2) \\
\cdots & \cdots & \cdots & \cdots & \cdots & \cdots & \cdots \\
\widehat{f}_{l1N} & \widehat{f}_{l1N}\widehat{f}_{11N} & \cdots & \widehat{f}_{l1N}\widehat{f}_{L1N} & \widehat{f}_{l1N}u_1(t_N) & \cdots & \widehat{f}_{l1N}u_P(t_N) \\
\widehat{f}_{l21} & \widehat{f}_{l21}\widehat{f}_{121} & \cdots & \widehat{f}_{l21}\widehat{f}_{L21} & \widehat{f}_{l21}u_1(t_1) & \cdots & \widehat{f}_{l21}u_P(t_1) \\
\widehat{f}_{l22} & \widehat{f}_{l22}\widehat{f}_{122} & \cdots & \widehat{f}_{l22}\widehat{f}_{L22} & \widehat{f}_{l22}u_1(t_2) & \cdots & \widehat{f}_{l22}u_P(t_2) \\
\cdots & \cdots & \cdots & \cdots & \cdots & \cdots & \cdots \\
\widehat{f}_{l2N} & \widehat{f}_{l2N}\widehat{f}_{12N} & \cdots & \widehat{f}_{l2N}\widehat{f}_{L2N} & \widehat{f}_{o2N}u_1(t_N) & \cdots & \widehat{f}_{l2N}u_P(t_N) \\
\cdots & \cdots & \cdots & \cdots & \cdots & \cdots & \cdots \\
\widehat{f}_{lS1} & \widehat{f}_{lS1}\widehat{f}_{1S1} & \cdots & \widehat{f}_{lS1}\widehat{f}_{LS1} & \widehat{f}_{lS1}u_1(t_1) & \cdots & \widehat{f}_{lS1}u_P(t_1) \\
\widehat{f}_{lS2} & \widehat{f}_{lS2}\widehat{f}_{1S2} & \cdots & \widehat{f}_{lS2}\widehat{f}_{LS2} & \widehat{f}_{lS2}u_1(t_2) & \cdots & \widehat{f}_{lS2}u_P(t_2) \\
\cdots & \cdots & \cdots & \cdots & \cdots & \cdots & \cdots \\
\widehat{f}_{lSN} & \widehat{f}_{lSN}\widehat{f}_{1SN} & \cdots & \widehat{f}_{lSN}\widehat{f}_{LSN} & \widehat{f}_{lSN}u_1(t_N) & \cdots & \widehat{f}_{oSN}u_P(t_N)
\end{bmatrix} \tag{16}
$$

3.1 Estimation of OTU trajectories and gradients: Bayesian Adaptive Penalized Counts Splines (BAPCS)

3.1.1 Model description

The objective of the Bayesian Adaptive Penalized Counts Splines (BAPCS) algorithm is to estimate OTU concentration trajectories and gradients from data consisting of time-series of noisy counts. Our formulation of the problem explicitly models differing numbers of sequencing reads between samples, overdispersion of counts, and irregular temporal sampling.

We assume that counts data follow the Negative Binomial Distribution (NBD), which we and others have previously employed for modeling microbial sequencing counts data [2, 3]:

$$Y_{lst} \sim \text{NBD}(g_{ls}(t), \epsilon_{ls}(t)) \tag{17}$$

Here, $g_{ls}(t)$ is a continuous-time trajectory function for the NBD mean for OTU l in subject s, and $\epsilon_{ls}(t)$ represents a time-varying dispersion parameter for the NBD.

For modeling the dispersion parameter, we use a method similar to that used by DESeq2 [4], but extended for time-series data. In the DESeq2 error model, the dispersion parameter was empirically found to be inversely proportional to counts for a given gene scaled by total counts plus an offset term. Our model for the dispersion parameter is:

$$\epsilon_{ls}(t) = \frac{a_0}{g_{ls}(t)/e^{\eta_{lt}}} + a_1 \tag{18}$$

Here, η_{lt} is a scaling factor for the total number of counts for the experiment (described in Section 3.1.5), and a_0 and a_1 are slope and offset parameters shared across experiments. Thus, in our formulation, the dispersion parameter varies over time, decreasing as the relative abundance of the OTU increases (as in the DESeq2 model). We assume uniform priors on a_0 and a_1 restricted to the interval $(0, 10^5)$ (an interval several orders of magnitude broader than the likely range of the parameters [4]). Note that in practice the likelihood will overwhelm the prior since the a_0 and a_1 parameters are shared across all samples and time-points, and will thus be estimated from a relatively large quantity of data.

We model $g_{ls}(t)$, the continuous-time trajectory function for the NBD mean for OTU l in subject s as follows:

$$g_{ls}(t) = e^{\mu_{ls}(t) + \eta_{st} - \kappa_{st} + \nu_{ls}} \tag{19}$$

Here η_{st} is a scaling factor for the total number of counts for the experiment, κ_{st} is a scaling factor for bacterial biomass, and ν_{ls} is an OTU and subject-specific scaling factor; the scaling factors are explained in Section 3.1.5.

We model μ_{ls} using cubic B-splines [5]:

$$\mu_{ls}(t) = \sum_{k=1}^{K} B_k(t)\phi_{lsk} \tag{20}$$

Here, $B_k(t)$ represent K B-spline basis functions (piece-wise polynomials) and ϕ_{lsk} represent the OTU and subject-specific spline coefficients for the trajectory.

B-splines provide an efficient and flexible framework for modeling time-series data, which we and others have demonstrated yield accurate predictions for large-scale longitudinal biological datasets [6]. The B-spline basis can be calculated using the cox-deBoor recursion formula [7]:

$$b_{k,1}(t) = \begin{cases} 1, & t_k \leq t < t_{k+1} \\ 0 & \text{otherwise} \end{cases} \tag{21}$$

$$b_{k,i}(t) = \frac{(t - t_k) b_{k,i-1}(t)}{t_{k+i-1} - t_k} + \frac{(t_{k+i} - t) b_{k+1,i-1}(t)}{t_{k+i} - t_{k+1}} \tag{22}$$

We use cubic B-splines (order 4), and thus $B_k(t) = b_{k,4}(t)$.

Each B-spline basis function has support (non-zero) values defined by the break-point times t_k. A challenge with B-spline methods is specifying the number of basis functions K and position of the break-points. These choices influence the amount of smoothing applied to the data. To overcome this challenge, we use a penalized spline framework [5] with an adaptive Bayesian regularization approach. Our approach chooses a relatively large number K of spline basis functions with placement of break-points uniformly (default of every 2 days), and then adaptively regularizes spline coefficients ϕ_{lsk} with support governed by adjacent break-points to encourage minimal changes in the trajectory over time unless larger deviations are warranted by the data. To achieve this, we use a hierarchical adaptive Bayesian fused lasso-style [8, 9] model.

We introduce variables ω^2_{lsj} that specify variance parameters providing shrinkage between spline coefficients governed by adjacent break-points (thus, temporally nearby regions.) We define the diagonal matrix Q_s of variance parameters on the spline coefficients as $Q_s = \text{diag}(\tau^2_{ls1}, \ldots, \tau^2_{lsK}, \omega^2_{ls1}, \ldots, \omega^2_{ls(K-d)})$. Here, τ^2_{lsj} represent individual variances on the spline coefficients ϕ_{lsk}. We assume $\tau^2_{lsj} = 10^2$, which are large variance values relative to the scale of the data (effectively standardized to mean zero and variance one in log space) and thus provide a relatively diffuse prior for the individual spline coefficients.

The matrix Υ provides the structure of individual variances on spline coefficients and regularization of spline coefficients governed by adjacent break-points:

$$\Upsilon = \begin{bmatrix} I_K \\ \mathbf{\Delta}_d \end{bmatrix} \tag{23}$$

Here, $\mathbf{\Delta}_d$ is the $K \times (K - d)$ d-order difference operator matrix, i.e., for a first order difference (the default setting):

$$\mathbf{\Delta}_1 = \begin{bmatrix} 1 & -1 & 0 & 0 & \cdots & 0 & 0 \\ 0 & 1 & -1 & 0 & \cdots & 0 & 0 \\ & & & & \cdots & & \\ 0 & 0 & 0 & 0 & \cdots & 1 & -1 \end{bmatrix} \tag{24}$$

We then have the following hierarchical probability model for spline coefficients ϕ_{lsk}:

$$\lambda^2_{lj} \sim \text{Gamma}(h_1, h_2) \text{ for } j = 1 \ldots K - d, \text{ where } d \text{ is the difference order} \tag{25}$$

$$\omega^2_{lsj} \sim \text{Exponential}(\lambda_{lj}) \tag{26}$$

$$\phi_{lsk} \sim \text{Normal}(0, \Upsilon^T Q_s \Upsilon) \tag{27}$$

Here, λ^2_{lj} are regularization parameters for OTU l and break-point j shared across subjects, allowing for borrowing of statistical strength across subjects in terms of the degree of smoothness of the trajectory in a temporal region (adjacent break-points). This approach allows the model to learn where major changes in the trajectory may occur (e.g., different degrees of regularization throughout the trajectory) across subjects. Note that our approach does not make the assumption that the trajectory values at each time-point have the same mean value across subjects. Such an assumption is likely to be overly restrictive for *in vivo* microbial dynamical systems in which subject-to-subject variability may be high and temporal synchronization may only occur with strong perturbations [10].

3.1.2 Model inference

We use a custom MCMC algorithm with Metropolis-Hastings (MH) and Gibbs updates as follows:

1. Sample the spline coefficients $\boldsymbol{\phi}$ conditional on the variances $\boldsymbol{\tau}^2$ and $\boldsymbol{\omega}^2$ using MH updates. We use an efficient MH update that we previously developed [3], which is based on a Generalized Linear Model (GLM) approximation for the NBD. Note that for the present model, it is not formally a GLM, given the dependency structure of the dispersion parameter (Equation 18). However, in practice, we found that the GLM approximation yields high acceptance rates. Also, to improve potentially poor initializations, we sample $\boldsymbol{\phi}$ from a linear model on the log transformed counts data for a small number of iterations during burn-in.

2. Sample variances $\boldsymbol{\omega}^2$ conditional on the spline coefficients $\boldsymbol{\phi}$ and regularization parameters $\boldsymbol{\lambda}$ using Gibbs updates from an inverse-Gaussian distribution as described in [11].

3. Sample $\boldsymbol{\lambda}$ conditional on variances $\boldsymbol{\omega}^2$, using Gibbs updates from a Gamma distribution as described in [9].

4. Sample the dispersion slope and offset parameters, a_0 and a_1, using MH updates. We assume independent log-normal moves with rejection of samples outside the support interval of $(0, 10^5)$.

For analysis of the datasets in the main manuscript, we used 10,000 MCMC samples with a burn-in of 2,000 samples, which we found to provide sufficient mixing.

3.1.3 Setting of hyperparameters and initializations

We set hyperparameters to achieve diffuse (high variance) priors. This is accomplished by estimating means and variances of prior distributions from data, and then setting the variance on the prior distribution to a significantly larger (inflated) value.

- Spline coefficients are initialized based on the log transformed counts data: $\boldsymbol{\phi}_{ls} \leftarrow \boldsymbol{B}_l^+(\log(Y_{ls}+1) - \eta_{st} + \kappa_{st} - \nu_{ls})$, where $\boldsymbol{B}_l^+$ is the pseudoinverse of the B-spline basis matrix.

- Variances on spline coefficients are set using a 1000X inflated estimate based on the log transformed counts data variance: $\tau^2_{slk} = 10^3 \cdot \text{median}_{sl}\Big(\text{Var}_t\{\log(Y_{lst}+1) - \eta_{st} + \kappa_{st} - \nu_{ls}\}\Big)$.

- Variances on spline coefficient differences are initialized based on the variance of the differenced log transformed counts data: $\omega^2_{lsj} \leftarrow \text{median}_{sl}\Big(\text{Var}_t\{\Delta_d(\log(Y_{lst}+1) - \eta_{st} + \kappa_{st} - \nu_{ls})\}\Big)$, where d is the difference order.

- Regularization parameters are initialized based on the log transformed counts data: $\lambda^2_{lj} \leftarrow 10^2/\text{median}_{sl}\Big(\mathbb{E}_t\{|\Delta_d(\log(Y_{lst}+1) - \eta_{st} + \kappa_{st} - \nu_{ls})))|\}\Big)^2$ (see [8, 11] for details on this form of initialization for the regularization parameters)

- Hyperparameters for the Gamma prior on regularization parameters: $h_1 = 10^{-6}$ and $h_2 = 10^6\lambda$, i.e., specifying a Gamma distribution with mean of the initial value of regularization parameters estimated from data and a large variance of $10^6 \cdot \lambda^2$.

- NBD dispersion offset and slope parameters: $a_1 \leftarrow 10^{-3}$ and $a_0 \leftarrow 10(1-a_1)e^{-\max_{st}\{\eta_{st}\}}$:, i.e., the initial estimate of a_1 set to a small value per DESeq2 [4] and the initial estimate of a_0 set such that a species with low abundance has a dispersion parameter of 10.

3.1.4 Computing trajectory and gradient estimates from samples

Summary estimates of OTU concentration values are calculated from the MCMC samples as:

$$\widehat{f}_{lst} = \text{median}_j\Big(e^{\sum_{k=1}^K B_k(t)\phi^{(j)}_{lsk} + \nu_{ls}}\Big) \tag{28}$$

Here, $\phi_{lsk}^{(j)}$ denotes MCMC sample j for the variable.

Summary estimates of OTU concentration gradient values are calculated from the MCMC samples as:

$$\widehat{f}'_{lst} = \text{median}_j \left(e^{\sum_{k=1}^{K} B_k(t)\phi_{lsk}^{(j)} + \nu_{ls}} \sum_{k=1}^{K} B'_k(t)\phi_{lsk} \right) \tag{29}$$

Here, $B'_k(t)$ denotes the derivative of B-spline basis function k.

3.1.5 Scaling factors

To account for differences in total numbers of sequencing reads across experiments, we compute a scaling factor for each sample:

$$\eta_{st} = \log(\sum_l Y_{lst}/\text{median}_{ij}(\eta_{ij})) \tag{30}$$

This factor takes into account the total number of sequencing reads for each sample, while rescaling by the median reads per sample to avoid numerical issues due to large numbers. See [3] for further details on this scaling method.

To account for the biomass in each sample, we first apply a trajectory estimation method to the biomass measurements. This estimation method is the same as that described for counts data above, except that we assume the log of biomass follows a normal distribution. The estimated biomass trajectory values, $\widehat{W}_{st}$, are then used to compute a scaling factor for each sample:

$$\kappa_{st} = \log(\widehat{W}_{st}/\text{median}_{ij}(\kappa_{ij})) \tag{31}$$

To avoid numerical issues due to large differences in the magnitudes of OTU trajectories, we compute scaling factors for each OTU and subject:

$$\nu_{ls} = \text{mean}_t(\log(Y_{lst} + 1)) \tag{32}$$

3.2 Bayesian gLV parameter estimation method 1: Bayesian adaptive lasso (BAL)

In this approach, we use an adaptive ℓ_1 or "lasso"-type regularization technique for the microbe-microbe interactions and perturbation effects.

3.2.1 Model definition

We assume the following regression model for our approach:

$$\widehat{f}'_{lst} \sim \text{Normal}(\alpha_l \widehat{f}_{lst} + \beta_{ll}\widehat{f}^2_{lst} + \sum_{j \neq l} \beta_{lj}\widehat{f}_{lst}\widehat{f}_{jst} + \sum_{i=1}^{P} \gamma_{li}\widehat{f}_{lst}u_i(t), \sigma_l^2) \tag{33}$$

We assume an OTU-specific variance σ_l^2 with an improper prior density $1/\sigma_l^2$. This prior for data variance is a popular choice for Bayesian linear regression models [12], and indeed was used in the seminal Bayesian lasso model publications [8, 11] as it yields a proper posterior with even relatively small datasets and reasonable settings for the lasso parameters. In practice, the effect of the prior on the data variance posterior is typically small, because many observations are available. For our model, σ_l^2 is estimated per OTU for all time-points in all subjects, i.e., 130 or 280 measurements for the *C. difficile* infection or probiotic cocktail datasets respectively, which result in negligible contributions from the prior.

We assume Normal priors with mean zero for the interaction and perturbation effect parameters:

$$\beta_{lj} \sim \text{Normal}(0, \tau_{lj}^2) \tag{34}$$

$$\gamma_{li} \sim \text{Normal}(0, \varrho_{lj}^2) \tag{35}$$

As in Section 3.1, we use a hierarchical model formulation to specify the Bayesian adaptive lasso (in this case for the variance parameters τ_{lj}^2 and ϱ_{lj}^2):

$$\lambda_{lj}^2 \sim \text{Gamma}(h_{\lambda 1}, h_{\lambda 2}) \tag{36}$$

$$\tau_{lj}^2 \sim \text{Exponential}(\lambda_{lj}) \tag{37}$$

$$\xi_{li}^2 \sim \text{Gamma}(h_{\xi 1}, h_{\xi 2}) \tag{38}$$

$$\varrho_{li}^2 \sim \text{Exponential}(\xi_{li}) \tag{39}$$

Here, λ_{lj}^2 and ξ_{li}^2 represent the regularization parameters for the microbe-microbe interactions and perturbation effects respectively.

We assume truncated Normal priors for the growth and self-interaction parameters (e.g., to enforce non-negative growth rates and negative self-interaction terms):

$$\alpha_l \sim \text{Normal}_{(+)}(\bar{\alpha}, \rho_{growth}^2) \tag{40}$$

$$\beta_{ll} \sim \text{Normal}_{(-)}(\bar{\beta}, \rho_{self}^2) \tag{41}$$

3.2.2 Model inference

Our objective is to infer the posterior distribution over the microbe-microbe interaction and perturbation effect coefficients. We use a custom MCMC algorithm with Gibbs sampling updates for all variables.

1. Sample interaction and perturbation effect variables β_{lj} (for $l \neq j$) and $\boldsymbol{\gamma}$, conditioned on the data and growth and self-interaction coefficients: via direct sampling from the multivariate Normal distribution.

2. Sample growth and self-interaction variables $\boldsymbol{\alpha}$ and β_{ll}, conditioned on the data and interaction and perturbation effect variables β_{lj} (for $l \neq j$) and $\boldsymbol{\gamma}$: via direct sampling from univariate truncated Normal distributions.

3. Sample interaction and perturbation effect variances τ_{lj} (for $l \neq j$) and $\boldsymbol{\varrho}$ conditioned on the interaction and perturbation effect variables β_{lj} (for $l \neq j$) and $\boldsymbol{\gamma}$ and regularization parameters λ_{lj} and $\boldsymbol{\xi}$: via direct sampling from inverse-Gaussian distributions (see [8, 9]).

4. Sample regularization parameters λ_{lj} and $\boldsymbol{\xi}$ conditioned on interaction and perturbation effect variances τ_{lj} (for $l \neq j$) and $\boldsymbol{\varrho}$: via direct sampling from Gamma distributions.

5. Sample OTU-specific variance σ_l^2: via direct sampling from Gamma distributions.

For analysis of the datasets in the main manuscript, we used 10,000 MCMC samples with a burn-in of 2,000 samples, which we found to provide sufficient mixing.

3.2.3 Setting of hyperparameters and initializations

We set hyperparameters to achieve diffuse (high variance) priors. In most cases, we accomplish this by estimating the mean and variance of the prior distribution from data, and then setting the variance on the prior distribution to a significantly larger (inflated) value.

- Growth, self-interaction, interaction and perturbation effect parameters: these are initialized from the linear regression $\widehat{F}_l' = X\boldsymbol{\theta}$ using the pseudoinverse solution, i.e., $\widehat{\boldsymbol{\theta}} = \widehat{F}_l' X^+$. We enforce positive growth and negative self-interaction parameters as $\widehat{\alpha} = |\widehat{\alpha}|$ and $\widehat{\beta}_{ll} = -|\widehat{\beta}_{ll}|$.

- Growth and self-interaction means $\bar{\alpha}$ and $\bar{\beta}$ and variance parameters ρ_{growth}^2 and ρ_{self}^2: these are set by taking the empirical means or variances of $\widehat{\boldsymbol{\theta}}$, with variances inflated by 10^4.

- Interaction and perturbation effect variances τ_{lj} (for $l \neq j$) and $\boldsymbol{\varrho}$: these are initialized by computing the empirical variances of $\widehat{\boldsymbol{\theta}}$ and inflating by 10^4.
- Regularization parameters $\boldsymbol{\lambda}$ and $\boldsymbol{\xi}$: these are initialized using the empirical estimates $\widehat{\boldsymbol{\theta}}$ derived as described above. We initialize $\boldsymbol{\lambda} \leftarrow 1/\Big(\mathbb{E}_l(\widehat{\beta}_{ll})\Big)^2$ and $\boldsymbol{\xi} \leftarrow 1/\Big(\mathbb{E}_{l,i}(\widehat{\gamma}_{li})\Big)^2$ (see [8] for details on this form of initialization for the regularization parameters).
- Hyperparameters for the Gamma priors on regularization parameters: $h_{\lambda 1} = h_{\xi 1} = 10^{-10}$ and $h_{\lambda 2} = 10^{10}\lambda$ (or $h_{\xi 2} = 10^{10}\xi$), i.e., specifying a Gamma distribution with mean of the initial value of regularization parameters estimated from data and a large variance of $10^{10} \cdot \lambda^2$ or $10^{10} \cdot \xi^2$.

3.3 Bayesian gLV parameter estimation method 2: Bayesian variable selection (BVS)

In this approach, we use a variable selection technique that directly models the presence or absence of microbe-microbe interactions or perturbation effects. Our model effectively learns a qualitative interaction network on the OTUs and perturbation effects, as well as a quantitative interaction and perturbation effects matrix.

3.3.1 Model definition

We assume the following regression model for our approach:

$$\widehat{f}'_{lst} \sim \text{Normal}(\alpha_l \widehat{f}_{lst} + \beta_{ll}\widehat{f}^2_{lst} + \sum_{j \neq l} z_{lj}\beta_{lj}\widehat{f}_{lst}\widehat{f}_{jst} + \sum_{i=1}^{P} q_{li}\gamma_{li}\widehat{f}_{lst}u_i(t), \sigma_l^2) \tag{42}$$

We assume an OTU-specific variance σ_l^2 with an improper prior density $1/\sigma_l^2$. This prior for data variance is a popular choice for Bayesian linear regression models [12], and indeed was used in the seminal Bayesian lasso model publications [8, 11] as it yields a proper posterior with even relatively small datasets and reasonable settings for the lasso parameters. In practice, the effect of the prior on the data variance posterior is typically small, because many observations are available. For our model, σ_l^2 is estimated per OTU for all time-points in all subjects, i.e., 130 or 280 measurements for the *C. difficile* infection or probiotic cocktail datasets respectively, which result in negligible contributions from the prior.

The variables z_{lj} and q_{li} represent binary indicators that select interaction and perturbation effect edges respectively. We assume $\boldsymbol{z}$ and $\boldsymbol{q}$ are Bernoulli distributed with parameters π_z and π_q:

$$z_{lj} \sim \text{Bernoulli}(\pi_z) \tag{43}$$

$$q_{li} \sim \text{Bernoulli}(\pi_q) \tag{44}$$

We place Beta priors on the Bernoulli distribution parameters:

$$\pi_z \sim \text{Beta}(b_{z1}, b_{z2}) \tag{45}$$

$$\pi_q \sim \text{Beta}(b_{q1}, b_{q2}) \tag{46}$$

We assume Normal priors for the interaction coefficients β_{lj} for $o \neq j$, and for the perturbation effect coefficients $\boldsymbol{\gamma}$:

$$\beta_{lj} \sim \text{Normal}(0, \rho^2_{interact}) \tag{47}$$

$$\gamma_{li} \sim \text{Normal}(0, \rho^2_{perturb}) \tag{48}$$

We assume truncated Normal priors for the growth and self-interaction parameters:

$$\alpha_l \sim \text{Normal}_{(+)}(\bar{\alpha}, \rho^2_{growth}) \tag{49}$$

$$\beta_{ll} \sim \text{Normal}_{(-)}(\bar{\beta}, \rho^2_{self}) \tag{50}$$

Finally, we place Normal priors on the growth and self-interaction mean parameters $\bar{\alpha}$ and $\bar{\beta}$, and inverse-Gamma priors on the variance parameters $\rho^2_{interact}$, $\rho^2_{perturb}$, ρ^2_{growth}, and ρ^2_{self}.

3.3.2 Model inference

Our objective is to infer the posterior distribution over edges in the microbe-microbe interaction and perturbation effect network, and the selected microbe-microbe interaction and perturbation effect coefficients. We use a custom MCMC algorithm with Gibbs sampling updates for all variables. The selection indicators z_{lj} and q_{li} are sampled using an efficient collapsed Gibbs update. This efficient update is possible, because we can compute the exact integral for marginalizing out interactions and perturbation effects.

The overall sampling scheme is as follows:

1. Sample interaction indicator and perturbation effect variables $\boldsymbol{z}$ and $\boldsymbol{q}$, conditioned on the data and growth and self-interaction coefficients: a collapsed Gibbs sampling step is used (described in detail below), iterating over all possible interactions and perturbation effects.

2. Sample interaction and perturbation effect variables β_{lj} (for $l \neq j$) and $\boldsymbol{\gamma}$, conditioned on the data and growth and self-interaction coefficients: via direct sampling from the multivariate Normal distribution.

3. Sample growth and self-interaction variables $\boldsymbol{\alpha}$ and β_{ll}, conditioned on the data and interaction and perturbation effect variables β_{lj} (for $l \neq j$) and $\boldsymbol{\gamma}$: via direct sampling from univariate truncated Normal distributions.

4. Sample indicator prior probabilities π_z and π_q, conditioned on indicator variables $\boldsymbol{z}$ and $\boldsymbol{q}$: via direct sampling from Beta distributions.

5. Sample growth and self-interaction mean parameters $\bar{\alpha}$ and $\bar{\beta}$, conditioned on growth and self-interaction parameters $\boldsymbol{\alpha}$ and β_{ll}: via direct sampling from univariate Normal distributions.

6. Sample variance parameters $\rho^2_{interact}$, $\rho^2_{perturb}$, ρ^2_{growth}, and ρ^2_{self}, conditioned on respective interaction, perturbation effect, growth or self-interaction parameters: via direct sampling from Gamma distributions.

7. Sample OTU-specific variance σ^2_l: via direct sampling from Gamma distributions.

For analysis of the datasets in the main manuscript, we used 25,000 MCMC samples with a burn-in of 2,500 samples, which we found to provide sufficient mixing.

Collapsed Gibbs sampling for indicator variables Let $X_l^{\{\boldsymbol{z},\boldsymbol{q}\}}$ denote the submatrix of design matrix X_l such that columns in X_l corresponding to indicator variables with values of zero are removed. Similarly, $\boldsymbol{\theta}^{\{\boldsymbol{z},\boldsymbol{q}\}}$ denotes the coefficient vector with entries corresponding to indicator variables with values of zero removed.

For collapsed Gibbs sampling, we need to compute the probability that interaction (or perturbation effect) i is or is not selected for OTU l. For brevity, we derive the case for an interaction variable being selected; the other cases are analogous. The marginal probability for this case is:

$$P(z_{li} = 1 \mid \boldsymbol{z}_{-li}, \boldsymbol{q}, \alpha_l, \beta_{ll}, \widehat{F}'_l, \sigma^2_l, \boldsymbol{\rho}, \bar{\alpha}, \bar{\beta}) \propto \\ P(z_{li} = 1) \int_{\boldsymbol{\theta}^{\{\boldsymbol{z}_{-li}, z_{li}=1, \boldsymbol{q}\}}_{-\alpha_l, \beta_{ll}}} P(\widehat{F}'_l \mid \boldsymbol{\theta}^{\{\boldsymbol{z}_{-li}, z_{li}=1, \boldsymbol{q}\}}, \sigma^2_l) P(\boldsymbol{\theta}^{\{\boldsymbol{z}_{-li}, z_{li}=1, \boldsymbol{q}\}} \mid \boldsymbol{\rho}, \bar{\alpha}, \bar{\beta}) \quad (51)$$

Here, marginalization is over all interaction and perturbation effect coefficients that are selected (e.g., $z_{lj} = 1$ or $q_{lj} = 1$), but *not* marginalized over the growth or self-interaction coefficients α_l and β_{ll}. The above integral can be written in closed form. Ignoring multiplicative terms that are identical between the case $z_{lj} = 1$ and $z_{lj} = 0$, we get:

$$P(z_{li} = 1 \mid \boldsymbol{z}_{-li}, \boldsymbol{q}, \alpha_l, \beta_{ll}, \widehat{F}'_l, \sigma^2_l, \boldsymbol{\rho}, \bar{\alpha}, \bar{\beta}) \propto \\ \pi_z \frac{\sqrt{\det(C)}}{\sqrt{\det(\Sigma_0)}} \mathrm{Normal}(\alpha_l, \beta_{ll}; m^{\{\alpha_l, \beta_{ll}\}}, C^{\{\alpha_l, \beta_{ll}\}}) \cdot e^{0.5 m^T C^{-1} m} \quad (52)$$

Here, $\Sigma_0 = \text{diag}(\rho^{\{\boldsymbol{z}_{li},\boldsymbol{q},z_{li}=1\}})$ is the prior covariance matrix, and m and C are the posterior mean and covariance matrix for the underlying Bayesian linear regression problem:

$$C = ((X_l^{\{\boldsymbol{z}_{li},\boldsymbol{q},z_{li}=1\}})^T X_o^{\{\boldsymbol{z}_{li},\boldsymbol{q},z_{li}=1\}}/\sigma_l^2 + \Sigma_0^{-1})^{-1} \tag{53}$$

$$m = C((X_l^{\{\boldsymbol{z}_{li},\boldsymbol{q},z_{li}=1\}})^T \widehat{F}_l'/\sigma_l^2 + \Sigma_0^{-1} m_0) \tag{54}$$

Here m_0 is the prior mean, i.e., $\bar{\alpha}$ for the growth parameters, $\bar{\beta}$ for the self-interaction parameters, and zero otherwise. The notation $m^{\{\alpha_l,\beta_{ll}\}}$ and $C^{\{\alpha_l,\beta_{ll}\}}$ denotes the subvector or submatrix of m and C restricted to the entries for α_l and β_{ll}.

3.3.3 Bayes factors

Bayes factors [13] provide a formal method for comparing two alternate models given evidence (data). For MDSINE, we use Bayes factors to assess the alternate models indicating the presence or absence of an interaction or perturbation effect given the data. For a given interaction between OTUs l and i the Bayes factor is calculated from J MCMC samples as:

$$\frac{\text{P}(z_{li}=1 \mid \widehat{F},\widehat{F}')\text{P}(z_{li}=0)}{\text{P}(z_{li}=0 \mid \widehat{F},\widehat{F}')\text{P}(z_{li}=1)} = \frac{\sum_j I(z_{li}^{(j)}=1)/J\int_{\pi_z}\text{P}(z_{li}=0 \mid \pi_z)}{\sum_j I(z_{li}^{(j)}=0)/J\int_{\pi_z}\text{P}(z_{li}=1 \mid \pi_z)} = \frac{\sum_j I(z_{li}^{(j)}=1)\cdot(b_{z1}+1)}{\sum_j I(z_{li}^{(j)}=0)\cdot(b_{z2}+1)} \tag{55}$$

Here, $I(\cdot)$ is the indicator function, and $z_{li}^{(j)}$ denotes the setting for z_{li} in MCMC sample j. Similarly, for perturbation effect p on OTU l, the Bayes factor is:

$$\frac{\sum_j I(q_{lp}^{(j)}=1)\cdot(b_{q1}+1)}{\sum_j I(q_{lp}^{(j)}=0)\cdot(b_{q2}+1)} \tag{56}$$

Per the Jeffreys scale for Bayes factors [13], we suggest considering interactions or perturbation effects with Bayes factors > 10 ("strong evidence.")

3.3.4 Setting of hyperparameters and initializations

We set hyperparameters to achieve diffuse (high variance) priors. In most cases, we accomplish this by estimating the mean and variance of the prior distribution from data, and then setting the variance on the prior distribution to a significantly larger (inflated) value.

- Growth, self-interaction, interaction and perturbation effect parameters: these are initialized using the mean of MCMC samples obtained from the Bayesian adaptive lasso algorithm.
- Interaction and perturbation effect indicator variables $\boldsymbol{z}$ and $\boldsymbol{q}$: these are initialized using MCMC samples from the Bayesian adaptive lasso algorithm. Interaction or perturbation effect indicators are set to 1 if their parameters from the MCMC samples have 95% credible intervals that do not contain zero.
- Growth and self-interaction means $\bar{\alpha}$ and $\bar{\beta}$, variance parameters $\rho^2_{interact}$, $\rho^2_{perturb}$, ρ^2_{growth}, and ρ^2_{self}, and hyperparameters for means and variance parameters: we first compute rough estimates of the growth, self-interaction, interaction and perturbation effect parameters via the linear regression $\widehat{F}_l' = X\boldsymbol{\theta}$ using the pseudoinverse solution, i.e., $\widehat{\boldsymbol{\theta}} = \widehat{F}_l' X^+$. We enforce positive growth and negative self-interaction parameters as $\widehat{\alpha} = |\widehat{\alpha}|$ and $\widehat{\beta}_{ll} = -|\widehat{\beta}_{ll}|$. The hyperparameters are then set by taking the empirical means or variances of $\boldsymbol{\theta}$, with variances inflated by 10^4. The parameters are then initialized to the means of their prior distributions.
- Interaction and perturbation effect indicator prior parameters and hyperparameters: we recommend two different default settings, depending on the intended application. For general predictive applications, we recommend $b_{z1} = 0.5$, $b_{z2} = 0.5$, $b_{q1} = 0.5$, and $b_{q2} = 0.5$. These hyperparameters correspond

to weak, agnostic priors (0.5 prior counts, equal probability of interaction/non-interaction or perturbation effect/non-perturbation effect). For applications in which a low false positive rate is desired we recommend $b_{z1} = L(L-1)$, $b_{z2} = 0.5$, $b_{q1} = L$ and $b_{q2} = 0.5$. This is a stronger prior, encoding the assumption that the expected total number of interactions or perturbation effects is 0.5 (e.g., not even one interaction or perturbation effect detected in the absence of evidence). For both cases, the prior parameters π_z and π_q are initialized using the means of their prior distributions.

4 Steady-state and stability estimation

The long-term behavior of the gLV system is determined by its steady-states (or fixed points). Let $[a_1, \ldots, a_L]$ denote the binarized profile of which OTUs are initially present, where a non-negative initial concentration for OTU l, $f_l(0) > 0$, implies $a_l = 1$, and $a_l = 0$, otherwise. The corresponding steady-state $\boldsymbol{f}^* = [f_1^*, ..., f_L^*]$ is then obtained by setting the time-derivative of Equation 1 (in absence of any applied perturbation) to zero, and solving the resulting system of linear equations:

$$\boldsymbol{f}^* = -\boldsymbol{\beta}^{-1}\boldsymbol{\alpha}, \tag{57}$$

where $\boldsymbol{\beta}$ is the microbial interactions matrix and $\boldsymbol{\alpha}$ is the vector of growth rates, both containing rows (and columns) of OTUs with positive initial profiles, i.e. for indices l with $a_l = 1$.

As we previously presented in [14], the asymptotic stability of $\boldsymbol{f}^*$ is described by the characterization of the spectrum of the corresponding Jacobian matrix of $g_l(f_1, \ldots, f_L) = f_l\left(\alpha_l + \sum_j \beta_{lj} f_j\right)$ evaluated at $\boldsymbol{f}^*$:

$$J_{\boldsymbol{f}^*} = \left(\frac{\partial g_l(\boldsymbol{f})}{\partial f_j}\right)_{l,j}\Bigg|_{\boldsymbol{f}=\boldsymbol{f}^*} = \begin{cases} \alpha_l + \beta_{ll} f_l^* + \sum_{j=1}^{L} \beta_{lj} f_j^* & \text{for } l = j \\ \beta_{lj} f_l^* & \text{for } l \neq j. \end{cases} \tag{58}$$

If all the eigenvalues of $J_{\boldsymbol{f}^*}$ have negative real part then $\boldsymbol{f}^*$ is asymptotically stable. We estimate the probability that a profile of OTUs $[a_1, \ldots, a_L]$ is asymptotically stable from K MCMC samples:

$$P_{\text{stable}}([a_1, \ldots, a_L]) = \frac{\sum_k I_{\text{RE}}(J_{\boldsymbol{f}^{*(k)}})}{K} \tag{59}$$

Here, $J_{\boldsymbol{f}^{*(k)}}$ denotes the Jacobian matrix for the steady-state value for MCMC sample k. $I_{\text{RE}}(\cdot) = 1$ if all eigenvalues for the matrix have negative real part and is equal to zero otherwise.

5 Benchmarking with simulated data

Ground-truth information is necessary for fully benchmarking the inferential and predictive capabilities of an algorithm. However, the availability of such data is currently too limited for microbial dynamical systems. Thus, we simulated data from an artificial microbial dynamical systems model to benchmark MDSINE. We attempted to mimic key features of real microbiome time-series data including noisy count-based measurements with limited sequencing depth, variability among subjects, limited temporal sampling resolutions, and a pathogen being introduced into a pre-existing microbiota. The simulated data was then use to evaluate several metrics of algorithm performance including: (a) accuracy of growth rates and interaction coefficients estimates, (b) accuracy of inference of presence/absence of microbial interactions (network structure), (c) accuracy of prediction of microbial concentrations over time given initial conditions not previously seen by the algorithm.

5.1 Data simulation procedure

We used the *C. difficile* infection dataset to obtain rough estimates of the scale and variability of growth rates, interaction coefficients, and initial concentrations of microbes. Estimates were obtained by solving the linear regression $\widehat{F}_l' = X\boldsymbol{\theta}$ using the pseudoinverse solution, i.e., $\widehat{\boldsymbol{\theta}} = \widehat{F}_l' X^+$. We enforced positive growth and negative self-interaction parameters as $\widehat{\alpha} = |\widehat{\alpha}|$ and $\widehat{\beta}_{ll} = -|\widehat{\beta}_{ll}|$. These estimates were then used to sample growth and interaction parameters θ_{sim} for a 10 OTU system as follows:

1. Growth rates: $\alpha_l \sim \text{Normal}_{(+)}(\bar{\alpha}, \sigma^2_{growth})$
2. Self-interaction parameters: $\beta_{ll} \sim \text{Normal}_{(-)}(\bar{\beta}, \sigma^2_{self})$
3. Interaction parameters: for each pair of OTUs l and j, we sampled whether an interaction occurs or not $z_{lj} \sim \text{Bernoulli}(\pi_z)$. We used $\pi_z = 0.20$, corresponding to a 20% probability of interaction between OTUs (roughly what was observed in the real dataset). If $z_{lj} = 0$, then we set $\beta_{lj} = 0$. If $z_{lj} = 1$, we sampled $\beta_{lj} \sim \text{Normal}(0, \sigma^2_{interact}/(\sum_i z_{li})^2)$. Here, we scale by the number of interactions, so that the carrying capacity for each OTU is approximately the same.

To obtain dynamical systems for testing, we required that all OTUs be present at steady-state and have coefficients of variation for their trajectories > 0.25 for 75% of initial concentrations tested (evaluated by generating 400 sets of initial conditions from a Normal distribution with mean and variance estimated from real data, and numerically integrating the gLV equations for each initial condition).

The parameters θ_{sim} were then used to generate simulated data for benchmarking as follows:

1. Sample initial concentrations for 10 subjects: we assumed normally distributed concentrations with mean and variance estimated from the *C. difficile* infection dataset. Sampled concentrations below 100 ($\approx$ 5% of the mean) were forced to 100, to ensure reasonably high initial concentrations for all OTUs.
2. Numerically integrate the gLV equations to obtain noise-free trajectories $f_{ls}(t)$, using the sampled initial conditions and parameters θ_{sim}: we integrated over 30 days, assuming 9 of the OTUs were introduced at time zero and the remaining OTU was introduced at day 10 (i.e., to simulate a pathogen being introduced into the system, as in the real data).
3. Sample noisy data from the gLV trajectories: we simulated both noisy counts data and biomass data from the gLV trajectories as follows. Biomass data was sampled as $W_{lst} \sim \text{Normal}(\sum_l f_{ls}(t), \sigma^2_{\text{biomass}})$, i.e., the noise-free biomass plus normally distributed noise. Count data was sampled from a Dirichlet-multinomial distribution (DMD) [15, 16]. The expected frequency parameters π_{lst} were set to the relative abundances of the underlying gLV trajectories, i.e., $\pi_{lst} = f_{ls}(t)/\sum_l f_{ls}(t)$. The dispersion parameter was set to 286 (estimated by maximum-likelihood fit using the MGLM toolkit [17] to the *Clostridium difficile* infection data to mimic error in real sequencing data.)

We investigated several regimens that varied the total number of counts (sequencing reads) per sample and the number of time-points sampled. We tested a range of sequencing depths between 200 to 25,000 reads to evaluate performance on an extremely limited sequencing depth up to realistic depths on the Illumina MiSeq platform. To evaluate the effects of different temporal sampling designs, we manually chose time-points to sample at for schemes with 27, 18, 12, or 8 data-points. Time-points were chosen to mimic real experimental designs for *in vivo* microbiome time-series datasets. Although daily sampling is ideal, due to economic and logistic constraints, many real-world longitudinal microbiome experimental designs employ limited and irregular temporal sampling [10]. These designs typically sample more frequently around events that are anticipated to cause major changes to the microbiota (e.g., initial colonization or introduction of a pathogen). To mimic these types of designs, for the simulated 27 time-point experiments, we sampled at days 0.5, 1, 2, 3, 4, 5, 6, 7, 8, 9, 10, 10.5, 11, 12, 13, 14, 15, 16, 18, 20, 21, 23, 24, 25, 27, 28, 30 (e.g., almost daily, to investigate a densely sampled time-series with only minimal irregular sampling). For the simulated 18 time-point experiments we sampled at days 0.50, 1, 2, 3, 4, 7, 9, 10, 10.50, 11, 12, 13, 14, 18, 21, 24, 28, 30 (e.g., approximately every 2 days with more frequent sampling during initial colonization and introduction of the pathogen at day 10). For the simulated 12 time-point experiments, we sampled at days 1, 2, 3, 7, 10, 11, 12, 14, 18, 21, 24, 30. For the simulated 8 time-point experiments, we sampled at days 1, 2, 7, 10, 14, 18, 24, 30.

5.2 Evaluation metrics

We evaluated simulated results for each of the algorithms implemented in the MDSINE package using the following metrics:

1. The root mean square error (RMSE) of the estimated growth rates compared to ground-truth growth rates.

2. The RMSE of the estimated interaction parameters compared to ground-truth interaction parameters.

3. The area under the receiver operator curve (AUC ROC) for presence/absence of interactions, for inferred interaction networks compared to the ground-truth network. We used the trapezoidal method for calculating the AUC ROC. For the BAL method, an interaction was called present if the $k\%$ credible interval for corresponding interaction coefficient β_{lj} did not contain zero. For the BVS method, an interaction was called present if the Bayes factor for the interaction was $> k$. For the MLCRR and MLRR methods, we employed a significance testing technique for ridge regression [18]. Briefly, the method computes a Student's t-statistic, with the strength of regularization (magnitude of the λ parameters) accounted for in the variance estimate.

4. The RMSE of predicted trajectories given only initial conditions compared to ground-truth trajectories. For this procedure, data was simulated for 10 subjects as described in Section 5.1. Then, data for one subject was held out, and the algorithms were trained on the remaining data. The trajectories for the held-out subject were then calculated by integrating the gLV equations using the system parameters θ estimated from the training data, and given initial conditions from the held-out subject.

The above metrics were computed on 400 samples for each of the regimens described in Section 5.1.

References

[1] Stein, R. R. *et al.* Ecological Modeling from Time-Series Inference: Insight into Dynamics and Stability of Intestinal Microbiota. *PLoS Comput Biol* **9**, e1003388 (2013).

[2] McMurdie, P. J. & Holmes, S. Waste Not, Want Not: Why Rarefying Microbiome Data Is Inadmissible. *PLoS Comput Biol* **10** (2014).

[3] Gerber, G. K., Onderdonk, A. B. & Bry, L. Inferring dynamic signatures of microbes in complex host ecosystems. *PLoS Comput Biol* **8**, e1002624 (2012).

[4] Love, M., Huber, W. & Anders, S. Moderated estimation of fold-change and dispersion for rna-seq data with deseq2. *Genome Biology* **15**, 550 (2014).

[5] Crainiceanu, C. M., Ruppert, D. & Wand, M. P. Bayesian analysis for penalized spline regression using winbugs. *Journal Of Statistical Software* **14** (2005).

[6] Bar-Joseph, Z., Gerber, G., Simon, I., Gifford, D. K. & Jaakkola, T. S. Comparing the continuous representation of time-series expression profiles to identify differentially expressed genes. *Proc Natl Acad Sci U S A* **100**, 10146–51 (2003).

[7] Rogers, D. & Adams, J. *Mathematical Elements for Computer Graphics* (McGraw-Hill, 1990).

[8] Park, T. & Casella, G. The bayesian lasso. *Journal of the American Statistical Association* **2008**, 681–686 (2008).

[9] Leng, C., Tran, M. & Nott, D. Bayesian adaptive lasso. *Annals of the Institute of Statistical Mathematics* **66**, 221–244 (2014).

[10] Gerber, G. K. The dynamic microbiome. *FEBS Lett* **588**, 4131–9 (2014).

[11] Casella, G., Ghosh, M., Gill, J. & Kyung, M. Penalized regression, standard errors, and bayesian lassos. *Bayesian Anal.* **5**, 369–411 (2010).

[12] Gelman, A., Carlin, J. B., Stern, H. S. & Rubin, D. B. *Bayesian Data Analysis* (Chapman & Hall, 2014).

[13] Kass, R. & Raftery, A. Bayes factors. *Journal of the American Statistical Association* **90**, 773–795 (1995).

[14] Stein, R. R. *et al.* Ecological modeling from time-series inference: Insight into dynamics and stability of intestinal microbiota. *PLoS Comput Biol* **9**, e1003388 (2013).

[15] Mosimann, J. On the compound multinomial distribution, the multivariate beta-distribution, and correlations among proportions. *Biometrika* **49**, 65–82 (1962).

[16] La Rosa, P. S. *et al.* Hypothesis testing and power calculations for taxonomic-based human microbiome data. *PLoS One* **7**, e52078 (2012).

[17] Zhang, Y., Zhou, H., Zhou, J. & Sun, W. Regression models for multivariate count data. *Journal of Computational and Graphical Statistics* (2016).

[18] Cule, E., Vineis, P. & De Iorio, M. Significance testing in ridge regression for genetic data. *BMC Bioinformatics* **12**, 372 (2011).
