# Supplementary figures and images for: MDSINE: Microbial Dynamical Systems INference Engine for microbiome time-series analyses
Source: Genome Biol. 2016 Jun 3;17:121. doi: 10.1186/s13059-016-0980-6 (PMC4893271; doi:10.1186/s13059-016-0980-6)

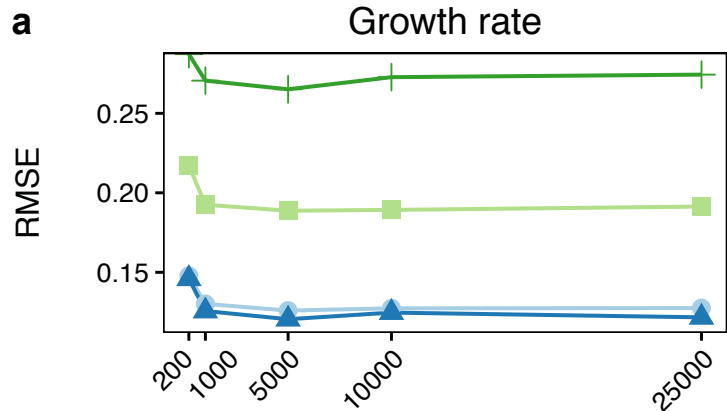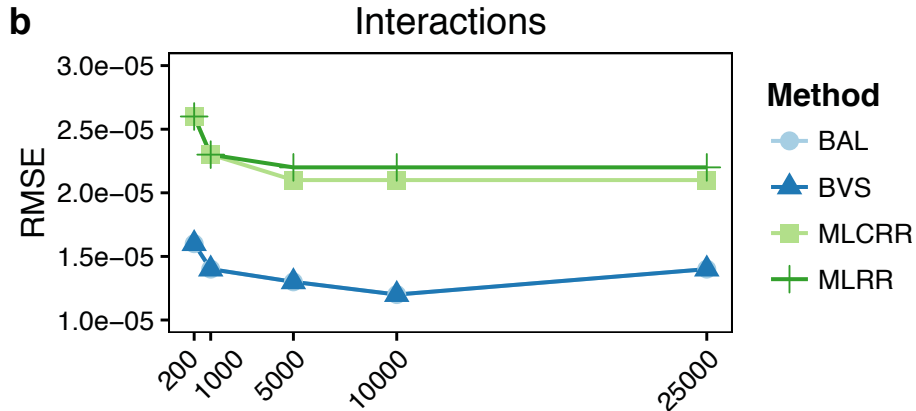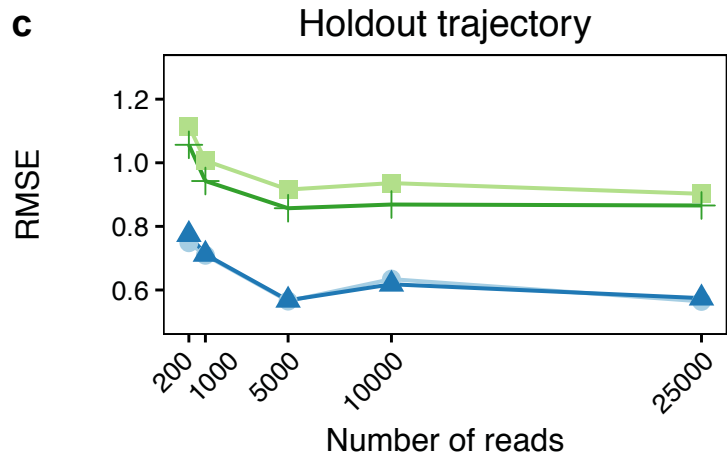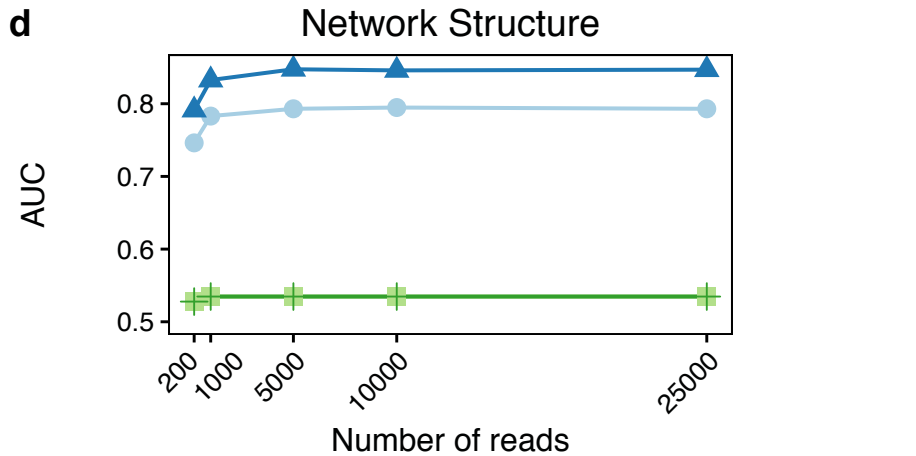

Supplement: Additional file 3: Figure S1. — Performance of the MDSINE inference algorithms on simulated data with different sequencing depths. Simulations assumed an underlying dynamical systems model with ten species observed over 30 days with 27 time points sampled and an invading species at day 10. Performance of the four MDSINE inference algorithms, maximum likelihood ridge regression (MLRR), maximum likelihood constrained ridge regression (MLCRR), Bayesian adaptive lasso (BAL), and Bayesian variable selection (BVS), were compared. Algorithm performance was assessed using four different metrics: (a) root mean-square error (RMSE) for microbial growth rates; (b) RMSE for microbial interaction parameters; (c) RMSE for prediction of microbe trajectories on held-out subjects given only initial microbe concentrations for the held-out subject; and (d) area under the receiver operator curve (AUC ROC) for the underlying microbial interaction network. Lower RMSE values indicate superior performance, whereas higher AUC ROC values indicate superior performance. (PDF 182 kb) [file 13059_2016_980_MOESM3_ESM.pdf]

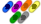

GnotoComplex

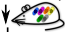

1 day

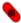

*C. difficile*

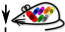

28

56

Supplement: Additional file 4: Figure S2. — Experimental design for Clostridium difficile infection studies in gnotobiotic mice. Five adult germfree mice were gavaged with 23 human commensal bacterial type strains, chosen to capitulate the phylogenetic diversity and key physiologic capabilities of a native gut flora (see “Methods” for details). After allowing 28 days for the commensal flora to establish, mice were gavaged with C. difficile spores and monitored for an additional 28 days. Fecal pellets were collected at days 0.75, 1, 2, 3, 4, 6, 8, 10, 14, 17, 21, 24, and 28 of the initial colonization and at days 0.75, 1, 2, 3, 4, 6, 8, 10, 14, 17, 21, 24, and 28 post-infection with C. difficile. Fecal samples were interrogated via high-throughput 16S rRNA sequencing to determine abundances of species and 16S rRNA qPCR using universal primers to estimate the total bacterial biomass present. After bioinformatics processing (see “Methods”), 13 strains were found to be consistently detectable in pre-infection stool samples. (PDF 87 kb) [file 13059_2016_980_MOESM4_ESM.pdf]

13 Clostridial strains

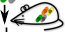

high fiber  
(5 weeks)

low fiber  
(2 weeks)

high fiber  
(2 weeks)

Supplement: Additional file 5: Figure S3. — Experimental design for probiotic stability studies in gnotobiotic mice. Seven adult germ-free mice were gavaged with 13 Clostridia strains from the VE202 probiotic cocktail [24]. Five mice were maintained on a standard high-fiber diet for 5 weeks, after which mice were switched to a low-fiber diet for 2 weeks and then switched back to the high-fiber diet for another 2 weeks; an additional two mice were inoculated with the same strains but were not subjected to the low-fiber dietary perturbation. Fecal pellets were collected at days 1–21 (daily), 23, 25, 27, 29, 31, 33, 35–60 (daily), 62, 63, and 65 for the five mice receiving the low-fiber dietary perturbation and at days 1–21 (daily), 23, 25, 27, and 29 for the two mice not receiving the perturbation. (PDF 42 kb) [file 13059_2016_980_MOESM5_ESM.pdf]

Normal, high-fiber diet

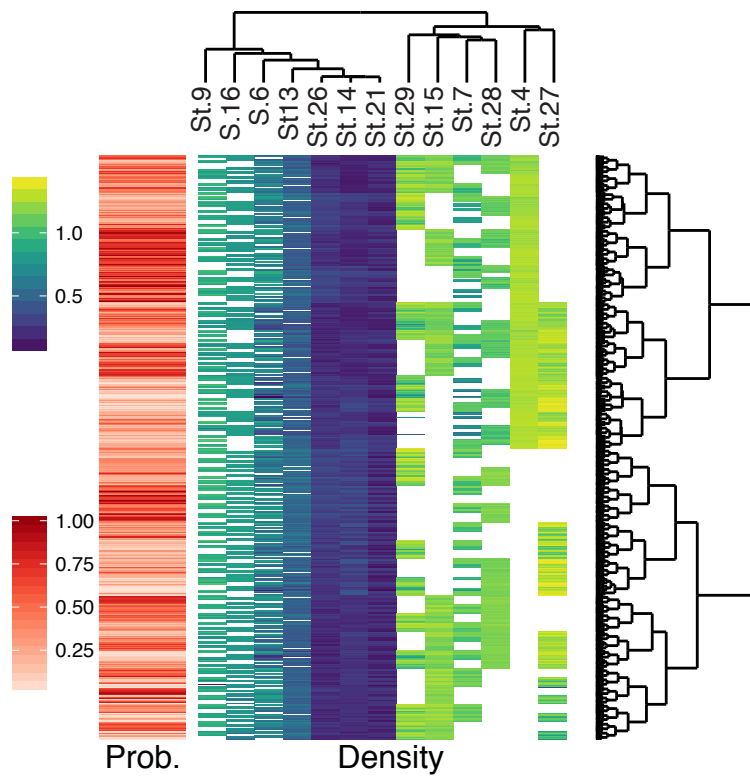

Low-fiber diet

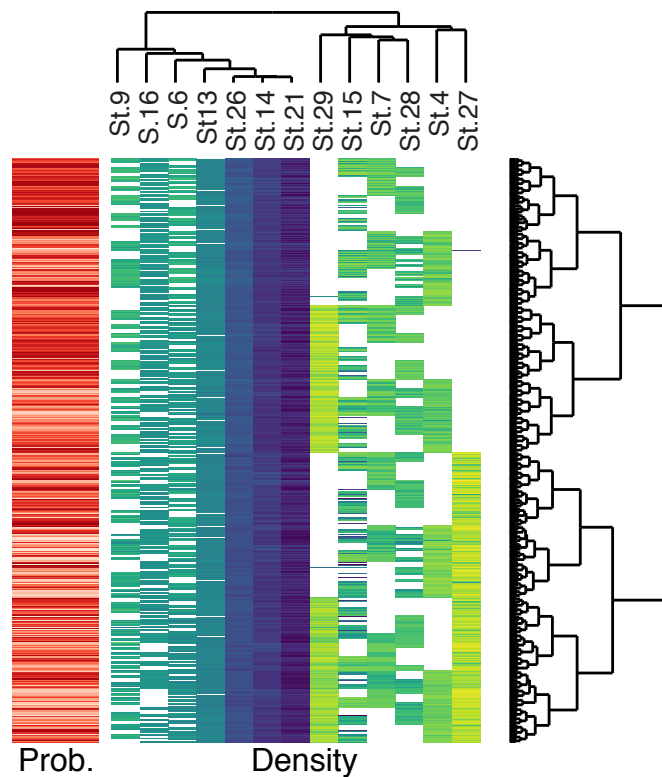

Supplement: Additional file 7: Figure S5. — Predicted stability and steady state concentrations (log10 ng strain DNA/μg total fecal DNA) for all combinations of the 13 Clostridia strains in mice fed either high-fiber (standard) or low-fiber diets in the probiotic stability experiment. Columns and rows were ordered using hierarchical clustering using Euclidean distance with Ward linkage. No significant differences were found in the predicted stable biodiversity profiles between the high-fiber and low-fiber dietary regimes (number of strains across all predicted stable states was not significantly different; Wilcoxon rank sum test p value = 0.096). (PDF 4845 kb) [file 13059_2016_980_MOESM7_ESM.pdf]

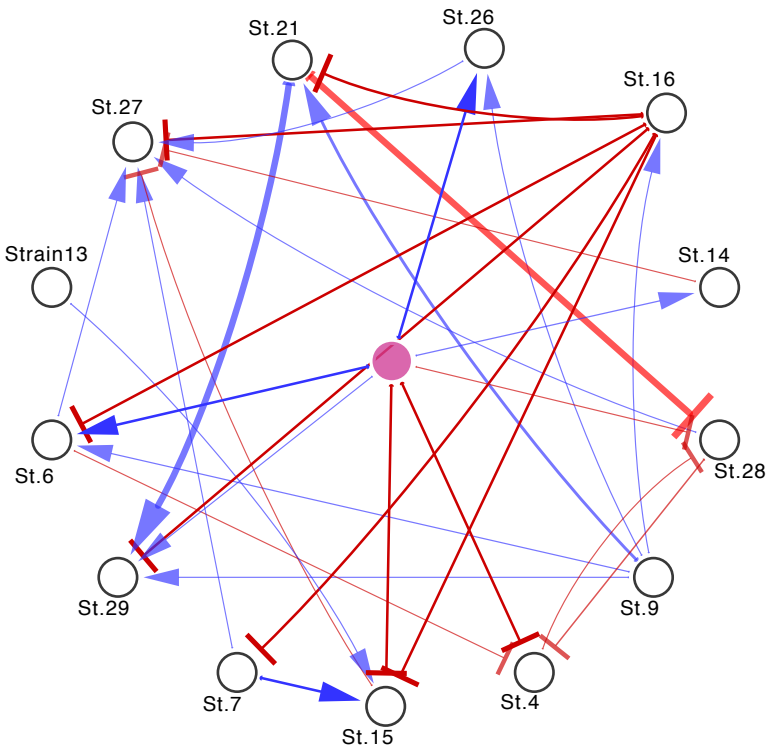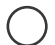

Strains

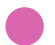

Low-fiber diet

Supplement: Additional file 8: Figure S6. — Directed microbe–microbe and microbe–perturbation effect network for the gnotobiotic mice probiotic stability experiment. Edge thickness denotes the magnitude of the evidence favoring presence of the interaction (Bayes factor). Only edges with strong evidence (Bayes factor ≥10) are displayed. (PDF 230 kb) [file 13059_2016_980_MOESM8_ESM.pdf]
