# Supplementary material for: MDSINE: Microbial Dynamical Systems INference Engine for microbiome time-series analyses
Source: Genome Biol. 2016 Jun 3;17:121. doi: 10.1186/s13059-016-0980-6 (PMC4893271; doi:10.1186/s13059-016-0980-6)
Supplement: Additional file 6: Figure S4. — Forecasts of microbial concentration trajectories for the gnotobiotic mice probiotic stability experiments. The forecasts were obtained using a hold-one-subject-out procedure. Briefly, MDSINE was run on all data from all but one of the mice (the held-out subject) and model parameters were inferred. Using the inferred model parameters (including for the perturbation) and the measured concentrations of the microbiota at an initial time point for the held-out mouse, the trajectories of the microbiota for the held-out mouse were then forecast for all the remaining time points; the procedure was repeated for each mouse in turn. Solid lines denote predicted trajectories and symbols denote actual data. (PDF 7552 kb) [file 13059_2016_980_MOESM6_ESM.pdf]

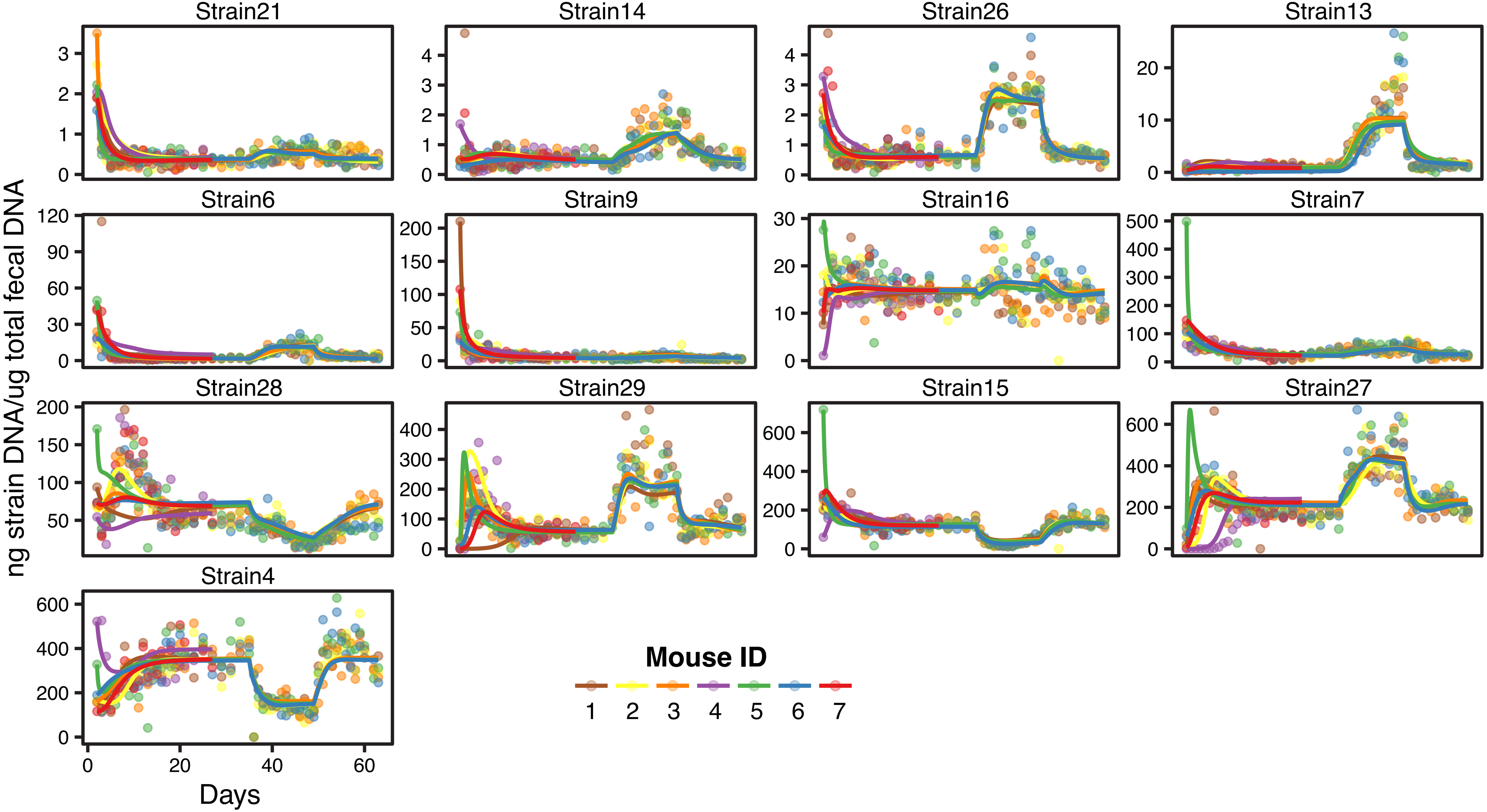

Strain21
Strain14
Strain26
Strain13
Strain6
Strain9
Strain16
Strain7
Strain28
Strain29
Strain15
Strain27
Strain4
ng strain DNA/ug total fecal DNA
Days
Mouse ID
1
2
3
4
5
6
7
